# 2-(5,6-Difluoro-3-oxo-2,3-dihydro-1*H*-inden-1-ylidene)-malononitrile and Naphtho[2,3-*b*]thiophene Diimide End-Capped Porphyrin Derivatives as Panchromatic Light-Harvesting Absorbers and Non-Fullerene Acceptors in Organic Solar Cells

**DOI:** 10.1021/acsomega.6c07199

**Published:** 2026-08-31

**Authors:** Manohar Reddy Busireddy, Yi-Chieh Chiu, Hung-Cheng Lin, Chain-Shu Hsu, Chia-Chih Chang

**Affiliations:** † Department of Applied Chemistry, 34914 National Yang Ming Chiao Tung University, 1001 University Road, Hsinchu 300093, Taiwan; ‡ Center for Emergent Functional Matter Science, National Yang Ming Chiao Tung University, 1001 University Road, Hsinchu 300093, Taiwan

## Abstract

Porphyrin-based nonfullerene acceptors (NFAs) are one of the most promising photoactive components in organic solar cells (OSCs) due to their excellent light-harvesting properties, featuring a high absorption coefficient of the Soret band in the ultraviolet–visible (UV–vis) region and an adjustable Q-band in the near-infrared (NIR) region, respectively. Moreover, the Acceptor−Donor−Acceptor (A–D–A) architecture is the most successful design strategy for constructing efficient NFAs. Herein, two porphyrin-based NFAs, named S-NPN-EH and T-NPN-OD, having naphtho­[2,3-*b*]­thiophene diimide (NTI) as the terminal acceptor units and porphyrin as a central core, are constructed with and without an acetylene linkage to elucidate the impact of linkage chemistry. For comparison, a 2-(5,6-difluoro-3-oxo-2,3-dihydro-1*H*-inden-1-ylidene) malononitrile (2FIC) end-capped porphyrin derivative, denoted as POR6−4FIC, with acetylene-bridged alkylthiophene as the π-spacers, is reported. The impact of the terminal NTI acceptor and acetylene linkage on structural, optoelectronic, and photovoltaic properties of S-NPN-EH and T-NPN-OD is systematically studied. The film-state UV–vis absorption of T-NPN-OD shows a more red-shifted spectrum with intensified Soret-/Q-bands in the NIR region over 1000 nm because acetylene bridges enhance intermolecular stacking and coplanarity compared to S-NPN-EH. In addition, T-NPN-OD exhibits a down-shifted lowest unoccupied molecular orbital (LUMO) energy level compared with S-NPN-EH, indicating that the S-NPN-EH-based device improves *V*_oc_ values. As expected, the reference POR6−4FIC shows strong red-shifted absorption over 1000 nm in the NIR region and down-shifted LUMO energy levels because of the strong intermolecular interactions between the donor and acceptors. The optimized device based on PTB7-Th/S-NPN-EH blend delivers a higher PCE of 2.19%, whereas the optimized PBDB-T:T-NPN-OD-based device shows a lower PCE of 1.01%. The reference PTB7-Th/POR6−4FIC-based device delivers a poor PCE of 0.32%. These results indicate that fine-balanced blend film morphology, well-matched energy levels, and solubility are critical for enhancing device performance.

## Graphical abstract

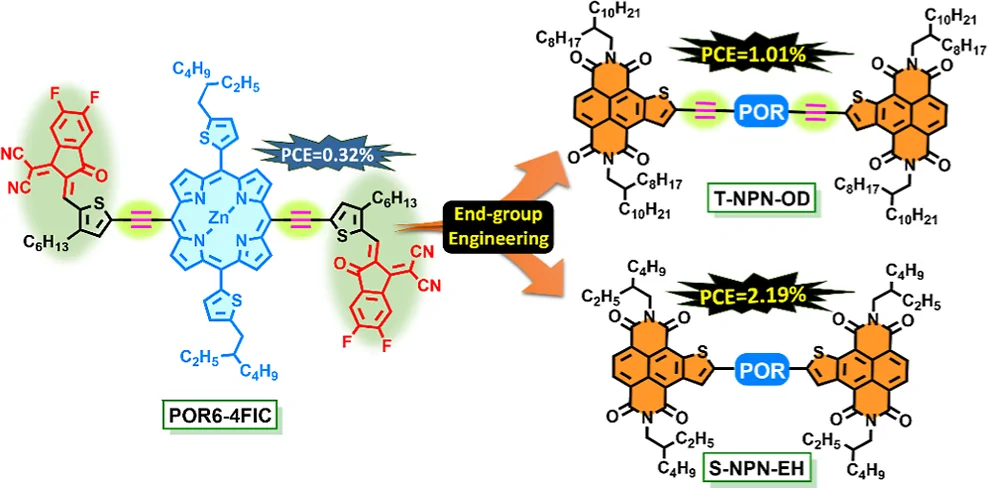

## 1 Introduction

Over the past two decades, the new generation of organic solar cells (OSCs) based on bulk heterojunction (BHJ) active layers has attracted much attention due to their potential for manufacturing lightweight, low-cost, semitransparent, and flexible solution-processed OSC devices.^1–7^ During the early development of OSCs, fullerene derivatives, especially phenyl-*C*_61_-butyric acid methyl ester (PC_61_BM) and [6,6]-phenyl-*C*_71_-butyric acid methyl ester (PC_71_BM), are dominant electron acceptors in OSCs because of their high electron affinity/electron mobility, charge transfer, and favorable nanoscale film morphology.^8–10^ As a result, devices using PCBM and suitable π-conjugated small molecules/polymer donors achieved power conversion efficiencies (PCEs) of over 10%.^11,12^ Nevertheless, fullerene derivatives possess several disadvantages, such as weak absorption in visible/near-infrared (NIR) regions, high synthetic cost, complex purification, and limited ability to tune their chemical structures and energy levels. Therefore, nonfullerene acceptors (NFAs) have attracted more attention due to their favorable attributes, such as easy synthesis and purification, cost-effectiveness in synthesis, strong absorption in visible/NIR regions, tunable chemical structures, and enhanced optoelectronic properties.^13–15^ Since the emergence of NFAs and highly efficient conjugated polymers, the PCE has improved significantly in recent years.^16–18^ In addition to the photoactive layer, interfacial layers play a crucial role in device performance, and recent advances in molecular and interfacial engineering have improved charge transport, energy-level alignment, and the overall photovoltaic performance.^19–21^ As a result, the PCEs of NFA-based devices have been recorded to exceed 18−20% in binary/ternary and tandem OSCs.^1,22–25^

At present, the high-performance NFAs have been constructed in two configurations: fused-/unfused-ring electron acceptors^13,19,25–29^ and rylene diimide (RyDI)-based electron acceptors (e.g., perylene diimide (PDI), naphthalene diimide (NDI), and its derivatives).^30–32^ RyDI-based derivatives are one of the widely investigated electron acceptors due to their structural versatility, strong electron affinities, high absorption coefficients, and excellent thermal stabilities. Moreover, the photovoltaic performance of RyDI-based NFAs has achieved over 9%.^32–36^ In early studies, PDI and fused PDI derivatives were widely investigated as NFAs in OSC active layers; however, due to the rigid/coplanar polycyclic aromatic skeleton and high crystallinity, which leads to excessive tendency of strong self-aggregation and forms micrometre-sized phase separation in the active layers, resulting in serious charge recombination and poor performance.^36,37^ To address this problem, several design strategies have been developed by the later OSC studies to reduce intermolecular interactions and excessive domain sizes of PDI-based NFAs, resulting in boosted PCEs.^38–41^ Among them, using different π-spacer/donor (D) units in a simple acceptor−donor−acceptor (A−D−A)-type configuration is one of the promising design strategies.^30,32,35^

Furthermore, NDI-based derivatives are extensively utilized as the A-units in polymer SCs, especially for the development of high electron mobility n-type polymers.^42–45^ Compared to PDI-based NFAs, NDI-based NFAs have not been studied extensively; there are only a few reports of NFAs using NDI and its derivatives as A-units.^33,34,41,46,47^ Thiophene-fused NDI/PDI A-units can enhance planarity to ensure red-shifted absorption and enhanced π–π stacking in the film state.^33–35^ For instance, Tajima and co-workers reported two naphtho­[2,3-*b*]­thiophene diimide (NTI)-based NFAs using anA−D−A-type configuration, which are constructed indaceno­[1,2-*b*:5,6-*b′*]­dithiophene (IDT) as a central D-unit and NTI moieties as terminal A-units, and the alkyl side chains were altered to optimize solvent processability and device performance.^33^ The resulting IDT-NTI-2EH and IDT-NTI-1HH showed a planar molecular backbone with strong π–π stacking and red-shifted absorption. Consequently, the IDT-NTI-2EH and IDT-NTI-1HH devices achieved PCEs of 9.07% and 5.05%, respectively, when blended with the PBDB-T polymer donor. The same group later reported two NFAs by replacing the central IDT moiety with spirobifluorene.^34^ The resulting SF-NTI-1HH showed a favorable face-on orientation with strong π–π stacking and delivered a PCE of 5.2% by blending with the PBDB-T polymer donor. Bo and co-workers constructed two thiophene-fused PDI (TFP)-based NFAs (denoted as CDT-TFP and C8X-TFP) with different alkyl chain-functionalized 4*H*-cyclopenta­[2,1-*b*:3,4-*b*′]­dithiophenes (CDTs) as the central D-unit.^32^ The resulting reg-PThE:CDT-TFP and reg-PThE:C8X-TFP devices delivered a PCE of 8.36% and 5.21%, respectively. Nevertheless, further enhancements of the photovoltaic performances in these molecules are limited by insufficient absorption in the near-infrared (NIR) region. Therefore, achieving NIR absorption of these NFAs by modifying the central D-moiety in A−D−A architecture is one of the plausible design strategies to improve the absorption window as well as the corresponding OSC device performance.^48–52^

On the other hand, porphyrin derivatives have attracted considerable attention to afford efficient small molecules/polymers for OSCs because of their strong light-harvesting ability in both ultraviolet–visible (UV–vis) and NIR regions and high stability, and their large/rigid planar structure can facilitate fine-tuning their energy levels and morphology.^53–59^ Porphyrin derivatives have been widely utilized in dye-sensitized solar cells as photosensitizers (DSSCs) and in perovskite solar cells as an interlayer, and the PCEs have exceeded 13 and 20%, respectively.^60–63^ In addition, porphyrin derivatives have also been extensively used as electron-donor/-acceptor materials for BHJ-OSC devices, and the corresponding device PCE values have reached over 10−12%.^64–67^ As for porphyrin-based NFAs, numerous examples have demonstrated the feasibility of employing porphyrin as the D-unit in constructing NFAs.^54–59,68–70^ In 2020, our lab also constructed A–D–A-type porphyrin-based NFAs using porphyrin as a central D-unit and conventional 2-(3-oxo-2,3-dihydro-1*H*-inden-1-ylidene)­malononitrile (IC)/halogenated IC moieties as the terminal A-units.^56^ The resulting NFAs showed strong Soret-/Q-band absorptions from 400 to 1000 nm, face-on orientation, resulting in decent photovoltaic performances. End-group acceptor modification and β/meso position engineering of porphyrins is a simple design strategy to tune the absorption and energy levels, resulting in the improvement of photovoltaic performances.^53–59,68–71^ To the best of our knowledge, there are no reports on using the NTI moiety on porphyrin-based NFAs despite many examples of conventional IC- and PDI/NDI-substituted porphyrin-based NFAs.^56–58,68–71^ Therefore, we anticipated that the development of NFAs with porphyrin/NTI D/A-moieties would be a worthwhile endeavor for affording NIR absorption.

Herein, we report two porphyrin-based NFAs, denoted as S-NPN-EH and T-NPN-OD, featuring an A−D−A architecture in which the terminal acceptor units are modified with NTI units, as shown in Figure 1. The constructed T-NPN-OD and S-NPN-EH have porphyrin as the central D-unit and NTI as the terminal A-units linked with and without an acetylene π-spacer, respectively. To elucidate the difference between NTI and conventional 2-(3-oxo-2,3-dihydro-1*H*-inden-1-ylidene)­malononitrile (IC)-based A-units, we also constructed a new porphyrin derivative POR6−4FIC as a benchmark, in which two 2-(5,6-difluoro-3-oxo-2,3-dihydro-1*H*-inden-1-ylidene) malononitrile (2FIC) are attached to a central porphyrin moiety as the terminal groups and alkylthiophene units as the π-spacers. The energy levels and UV absorption of the aforementioned compounds were characterized. The normalized absorption of T-NPN-OD and POR6−4FIC shows two pronounced, distinctive absorption bands (Soret-/Q-bands) in the solution state in comparison with S-NPN-EH. Notably, T-NPN-OD and POR6−4FIC show stronger red-shifted absorption over 900 nm in the film state than that of the S-NPN-EH film state. Furthermore, the lowest unoccupied molecular orbital (LUMO) energy level of S-NPN-EH is elevated, leading to an enhancement of the *V*_oc_. Moreover, NTI end-capped S-NPN-EH and T-NPN-OD molecules exhibit higher thermal stability than the 2FIC end-capped POR6−4FIC. Due to complementary absorptions and good alignment of energy levels, inverted OSC devices were fabricated using PTB7-Th as a donor for S-NPN-EH and POR6−4FIC and PBDB-T as a donor for T-NPN-OD devices. As a result, the optimal S-NPN-EH-based device delivers a higher PCE of 2.19% compared with T-NPN-OD- (PCE of 1.01%) and POR6−4FIC-based devices (PCE of 0.32%).

**Figure 1:**
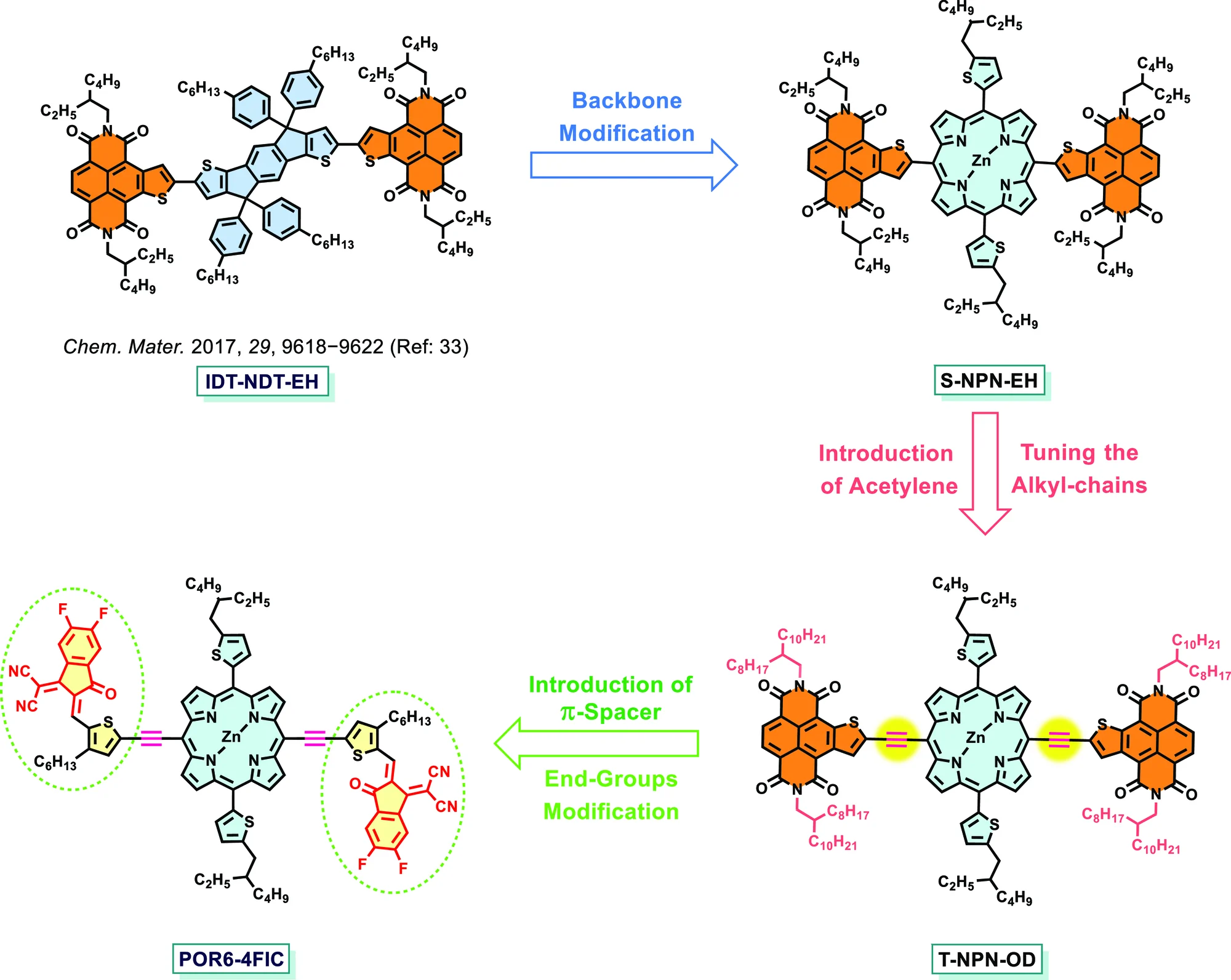
Design strategy and chemical structures of IDT-NDT-EH, S-NPN-EH, T-NPN-OD, and POR6−4FIC.

## 2 Experimental Section

### 2.1 Materials and Instrumentation

Unless otherwise specified, all chemicals/reagents were received from different commercial sources and used directly without any further purification. Synthetic details and characterization data are included in the Supporting Information. ^1^H NMR and ^13^C NMR spectra of all the molecules were recorded using Varian 400 MHz and VNMRS-600 NMR spectrometers. Mass spectral data of all compounds were recorded using JEOL instruments (model: JMS-T200GC AccuTOF GCx; source mode: field desorption (FD)). Under a nitrogen atmosphere, differential scanning calorimeter (DSC) and thermogravimetric analysis (TGA) were measured on a TA Q200 Instrument and a PerkinElmer Pyris, respectively. The UV–vis absorption spectra of all target compounds were measured by an HP8453 UV–vis spectrophotometer. For measuring the energy levels of all compounds, electrochemical cyclic voltammetry (CV) was conducted on a CH Instruments Model 611D. A carbon glass, standard Ag/Ag^+^ electrode, and platinum (Pt) wire were used as a working electrode, reference electrode, and counter electrode, respectively. A solution of 0.1 M tetrabutylammonium hexafluorophosphate (Bu_4_NPF_6_) in dichloromethane (DCM) was used as the electrolyte. In order to calibrate the oxidation and reduction curves, we used ferrocene as an internal reference standard.

### 2.2 Inverted OSC Device Fabrication for NFAs

The solution-processed inverted OSC device configuration of ITO/ZnO/active layer/MoO_3_/Ag was used in this study for measuring all the molecules’ photovoltaic performances. The inverted OSC devices were fabricated as follows: under ultrasonic irradiation, the ITO/glass substrates were cleaned sequentially in aqueous detergent water, deionized water, acetone, and isopropanol for 15 min (each one), followed by UV–ozone treatment for 25 min. The ZnO precursor solution was prepared according to our previous procedures.^18^ On top of the precleaned ITO/glass substrates, 40 μL of ZnO precursor solution was spin-coated at 4000 rpm/30 s and then thermally annealed at 180 °C for 30 min. For the preparation of the active layers, PTB7-Th:S-NPN-EH (a weight ratio of 1:1.2 and a total concentration of 17.6 mg/mL) was dissolved in 1,2-dichlorobenzene, and 1% pyridine as an additive was added. PBDB-T:T-NPN-OD (a weight ratio of 1:1 and a total concentration of 16.0 mg/mL) was dissolved in chlorobenzene, and PTB7-Th:POR6−4FIC (a weight ratio of 1:1.2 and a total concentration of 17.6 mg/mL) was dissolved in THF. All the resulting active layer solutions were stirred at 60 °C for 10 h before the deposition of active layers. Subsequently, on top of the ZnO/ITO/glass substrates, 40 μL of active layers was spin-coated at 1200 rpm/60 s. The resulting PTB7-Th:S-NPN-EH, PBDB-T:T-NPN-OD, and PTB7-Th:POR6−4FIC substrates were annealed at 130, 100, and 130 °C for 10 min, respectively. Finally, 7 nm of MoO_3_ and 100 nm of Ag were deposited on top of the active layers at 2.1 × 10^−7^ mbar base pressure using a thermal evaporation chamber. The measured device areas of the active layers were 0.04 cm^−2^. The current−voltage (*J−V*) curves of all devices were measured using a computer-controlled Keithley 2400 Source Meter under simulated AM 1.5G illumination of 100 mW cm^−2^ (1 sun) under air.

## 3 Results and Discussion

### 3.1 Synthesis and Thermal Properties

The chemical synthesis pathways of porphyrin-based NFAs S-NPN-EH, T-NPN-OD, and POR6−4FIC are outlined in Schemes 1 and S1. The details of experimental procedures and characterizations are provided in the Supporting Information. All the intermediate compounds 5b, 6a, T4, and P1 were prepared from the modified procedures of previously reported methods.^33,56^ Compound P2 was synthesized from compound P1 by bromination using *N*-bromosuccinimide (NBS). The intermediate P4 was prepared from compound P2 by a Sonogashira reaction with trimethylsilylacetylene, followed by the deprotection of the trimethylsilyl group in the presence of tetra-*n*-butylammonium fluoride. The target compound T-NPN-OD was prepared from compound P4 via a Sonogashira reaction with compound 5b. Subsequently, S-PNP-EH is synthesized via Pd­(PPh_3_)_4_ catalyzed Stille cross-coupling of compound P2 with compound 6a. Finally, the reference target compound POR6−4FIC was synthesized from compound P2 through a Sonogashira reaction with compound T4, followed by condensation with 2-(5,6-difluoro-3-oxo-2,3-dihydro-1*H*-inden-1-ylidene)­malononitrile (2FIC). All the intermediate compounds and final target compounds were fully characterized by ^1^H nuclear magnetic resonance (NMR), ^13^C NMR, and high-resolution mass spectroscopy (HRMS), and the corresponding spectral data are presented in the Supporting Information (Figures S1–S26). All three target compounds are soluble in all organic solvents such as chloroform (CHCl_3_), tetrahydrofuran (THF), chlorobenzene, etc. To measure the thermal stabilities of S-NPN-EH, T-NPN-OD, and POR6−4FIC, thermogravimetry analysis was conducted, and the thermograms are depicted in Figure S27a. All compounds exhibit good thermal stability; S-NPN-EH and T-NPN-OD show superior thermal stability in comparison to POR6−4FIC. The thermal decomposition temperature (*T*_d5%_) of S-NPN-EH, T-NPN-OD, and POR6−4FIC was 383, 396, and 336 °C, respectively. Furthermore, differential scanning calorimetry (DSC) was employed to characterize the melting and crystalline characteristics of S-NPN-EH, T-NPN-OD, and POR6−4FIC. The corresponding heating/cooling thermograms are presented in Figure S27b–d. The S-NPN-EH and POR6−4FIC show no obvious endothermic/exothermic peaks in their heating/cooling cycles; T-NPN-OD exhibits both endothermic/exothermic peaks corresponding to their melting (*T*_m_)/crystallization (*T*_c_) temperatures in heating/cooling cycles. The obtained *T*_m_/*T*_c_ peaks of T-NPN-OD are 235/201 °C, suggesting enhanced intermolecular interactions and molecular aggregations.

**Scheme 1:**
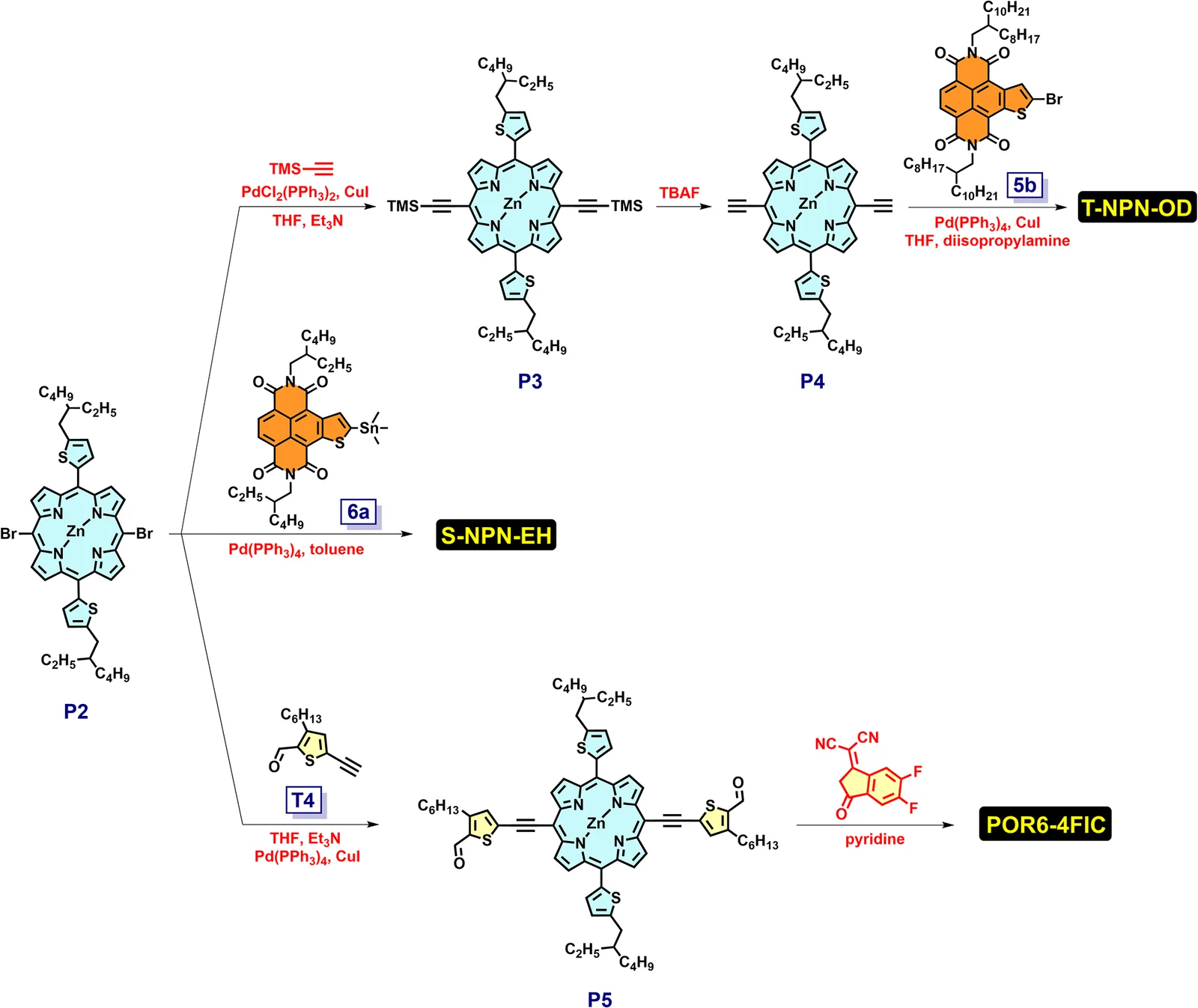
Synthetic Route of S-NPN-EH, T-NPN-OD, and POR6-4FIC

### 3.2 Optical and Electrochemical Properties

The UV–vis absorption spectra of S-NPN-EH, T-NPN-OD, and POR6−4FIC were measured in dilute THF solutions and film states. The corresponding UV–vis plots are depicted in Figure 2, and the data are summarized in Table 1. In the solution state (Figure 2a), T-NPN-OD exhibits two distinct absorption bands in the range of 390−620 nm and 620−880 nm, which correspond to the Soret- and Q-bands of porphyrin molecules, respectively.^54–58^ The stronger Soret-band absorptions are ascribed to the π–π* transition of the porphyrin conjugate backbone and the enhanced Q-band absorptions caused by intramolecular charge transfer (ICT) between the porphyrin donor unit and terminal NTI acceptor units.^54–58^ Interestingly, S-NPN-EH displays an intense Soret absorption band in the short wavelength region between 400 and 500 nm, while there is no strong absorption band at the long wavelength region, indicating weaker ICT characteristics between the porphyrin and NTI moieties. The reference POR6−4FIC shows very strong Soret-/Q-band absorptions in both short and long wavelength regions due to the strong withdrawing nature of the 2FIC terminal acceptors hanging in POR6−4FIC, which enhances the ICT characteristic peaks. These results indicate that T-NPN-OD and POR6−4FIC molecules show a strong planar geometry because the presence of acetylene π-bridges restricts the rotational freedom, which enhances the ICT behavior between donor/acceptor compared with the S-NPN-EH, in the solution state.

**Figure 2:**
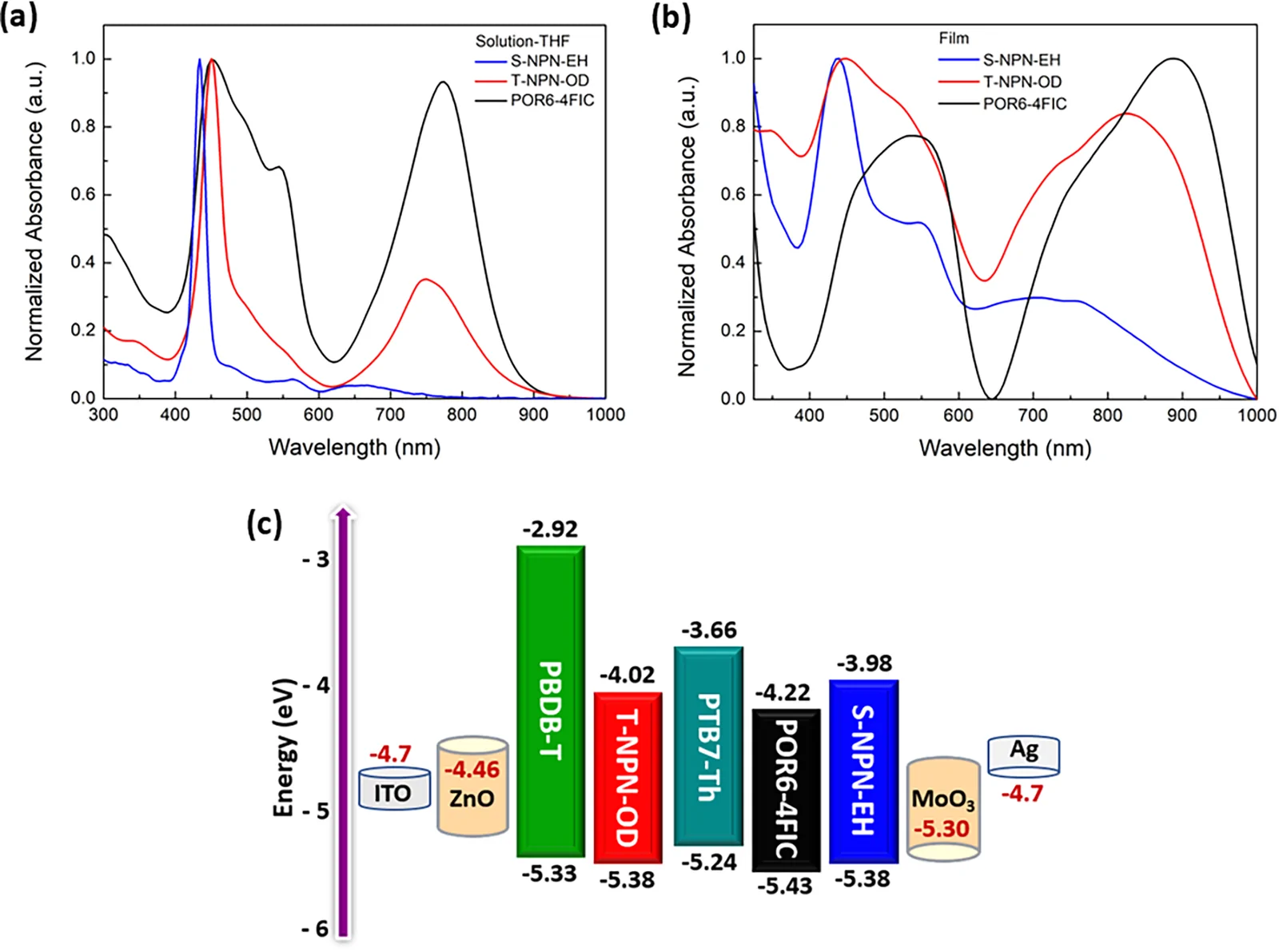
UV–vis absorption spectra of S-NPN-EH, T-NPN-OD, and POR6−4FIC in (a) THF solution and (b) film state. (c) HOMO and LUMO energy levels of all materials.

**Table 1:** Optical and Electrochemical Properties of S-NPN-EH, T-NPN-OD, and POR6-4FIC

materials	λ_max_^sol^ (nm)^a^	λ_max_^film^ (nm)^b^	λ_onset_^film^ (nm)^c^	λ_gap_^opt^ (eV)^d^	E_HOMO/LUMO_^DFT^ (eV)^e^	*E*_gap_^DFT^ (eV)^f^	E_HOMO/LUMO_^CV^ (eV)^g^	*E*_gap_^CV^ (eV)^h^
S-NPN-EH	435	440,760	879	1.41	−5.43/−3.44	1.99	−5.38/−3.98	1.40
T-NPN-OD	452, 747	449, 818	939	1.32	−5.31/−3.58	1.73	−5.38/−4.02	1.36
POR6−4FIC	450, 775	540, 890	969	1.28	−5.35/−3.57	1.78	−5.43/−4.22	1.21

a Maximum absorption wavelength in the THF solution state.

b Maximum absorption wavelength in the film state.

c Onset absorption wavelength in the film state.

d Optical band gap estimated from λ_gap_^opt^ = 1240/λ_onset_^film^ in the film state.

e HOMO/LUMO energy levels were calculated from DFT.

f Energy gap was calculated from DFT.

g HOMO/LUMO energy levels were calculated from CV.

h Energy gap was calculated from CV.

As shown in Figure 2b, the thin film absorption bands of all three NFA molecules show red-shifted absorption in short/long wavelength regions when compared to those in solution, which is due to the more ordered molecular packing in the solid state.^54–58^ Specifically, the two absorption band intensities of T-NPN-OD and reference POR6−4FIC are stronger, indicating stronger intermolecular interactions and aggregation in the film state. On the other hand, the S-NPN-EH shows a stronger Soret-band absorption intensity than the Q-band absorption intensity, which indicates weaker intermolecular packing and aggregation in the film state. Notably, the Q-band absorptions of T-NPN-OD and reference POR6−4FIC from 630 to 1000 nm significantly improved, indicating stronger intermolecular π–π stacking in the film state compared to the solution state, whereas the film state Q-band absorption of S-NPN-EH from 600 to 920 nm is also enhanced compared to the solution state. However, the Q-band absorption of S-NPN-EH is weaker compared with the T-NPN-OD and reference POR6−4FIC Q-band absorptions. Furthermore, the optical band gaps are calculated from the film state onset absorption spectra to be 1.41 eV for S-NPN-EH, 1.32 eV for T-NPN-OD, and 1.28 eV for POR6−4FIC. These results reveal that acetylene π-bridges enhance the planarity and decrease steric hindrance of the molecular geometry, leading to improved intermolecular π–π packing interactions in the film state.

To further calculate the HOMO/LUMO energy levels of S-NPN-EH, T-NPN-OD, and POR6−4FIC, cyclic voltammetry (CV) is performed. The obtained oxidation/reduction plots are illustrated in Figure S28. The HOMO energy levels are calculated from the equation of HOMO = −(4.8 eV + (*E*_ox_ − *E*_ref_)), and the LUMO energy levels are calculated from the equation of LUMO = −(4.8 eV + (*E*_red_ − *E*_ref_)), and the data are summarized in Table 1. The calculated HOMO/LUMO energy levels of S-NPN-EH, T-NPN-OD, and POR6−4FIC are −5.38/−3.98, −5.38/−4.02, and −5.43/−4.22 eV, with their band gaps of 1.40, 1.36, and 1.21 eV, respectively. From CV results, S-NPN-EH exhibits up-shifted LUMO energy levels compared with T-NPN-OD and POR6−4FIC, which improves the *V*_oc_ values.

### 3.3 Theoretical Calculation Results

To understand the structure properties and energy levels of the S-NPN-EH, T-NPN-OD, and POR6−4FIC, density functional theory (DFT) calculations are performed. In this study, S-NPN-EH, T-NPN-OD, and POR6−4FIC are optimized under B3LYP functionals with the 6-311G­(d,p)­basis set using the Gaussian 09 suite.^18^ In addition, the long alkyl chains are shortened to methyl groups to facilitate computational analysis. The optimized structures of S-NPN-EH, T-NPN-OD, and POR6−4FIC, along with their corresponding dihedral/torsion angles and bond lengths, are demonstrated in Figure S29. The optimized geometries reveal that T-NPN-OD and POR6−4FIC display a higher degree of planarity due to the acetylene π-bridge in comparison to S-NPN-EH, leading to an enhanced ICT character. In addition, the acetylene π-bridges decrease the steric crowding between porphyrin and terminal NTI units, which enhances the planarity and π-π stacking, leading to red-shifted absorption.^41^ Furthermore, the HOMO/LUMO energy level iso-surface plots of the Frontier molecular orbitals (FMOs) for S-NPN-EH, T-NPN-OD, and POR6−4FIC are presented in Figure 3. As illustrated, the HOMO is primarily localized on the central core porphyrin unit, whereas the LUMO is mainly distributed over the terminal acceptor units for all three molecules. The HOMO/LUMO energy levels are calculated to be −5.43/−3.44, −5.31/−3.58, and −5.35/−3.57 eV for S-NPN-EH, T-NPN-OD, and POR6−4FIC, respectively, which slightly deviate from the trend of the HOMO/LUMO energy levels obtained from CV measurement. This can be attributed to the fact that the DFT calculation was performed in vacuum. More importantly, structural planarity induced by acetylene linkage was observed.

**Figure 3:**
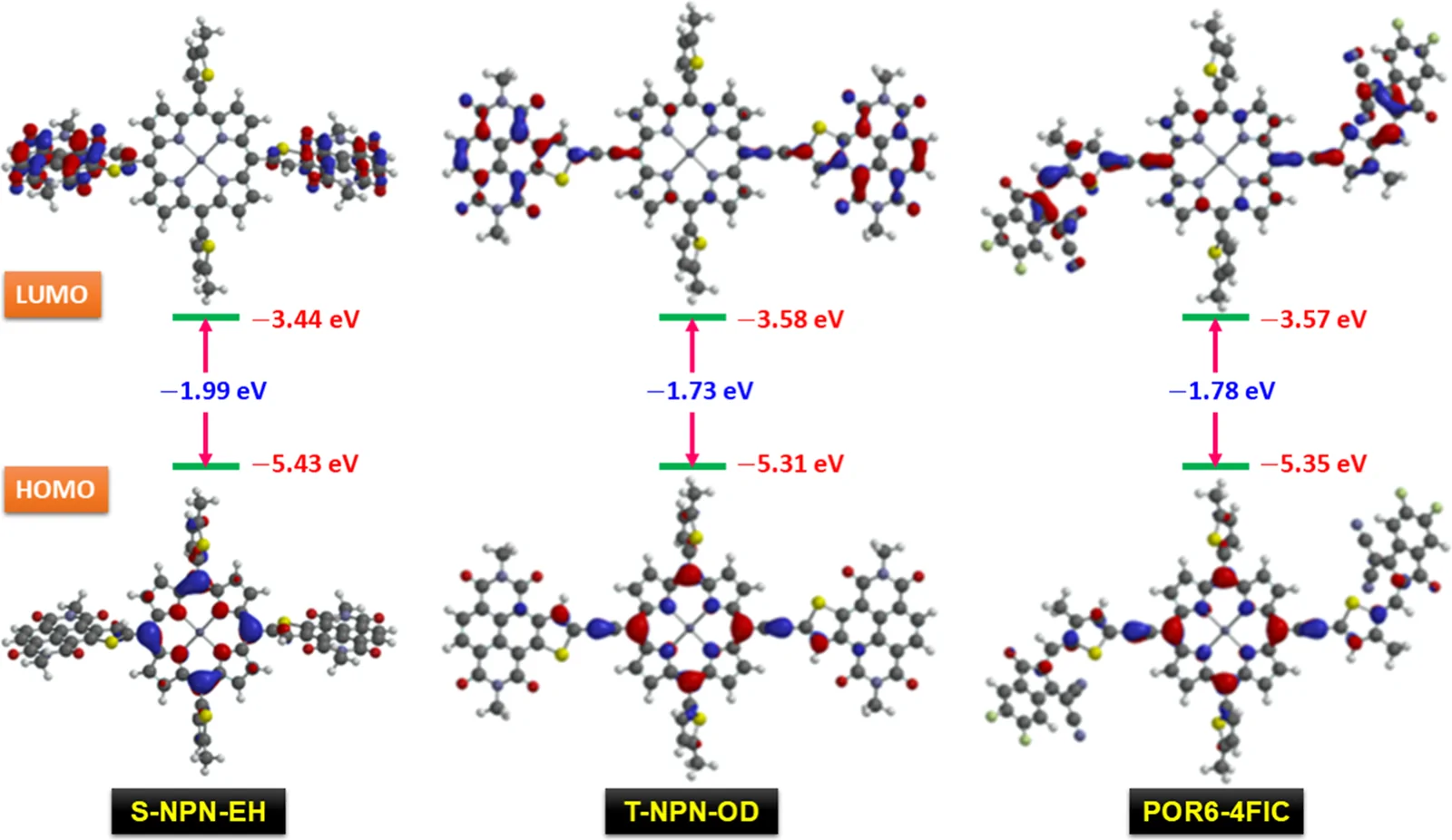
FMO distribution and HOMO/LUMO energy levels of S-NPN-EH, T-NPN-OD, and POR6−4FIC.

### 3.4 Photovoltaic Properties

The effect of terminal NTI acceptors on the photovoltaic performances of S-NPN-EH, T-NPN-OD, and POR6−4FIC is measured in an inverted OSC device with a configuration of indium tin oxide (ITO)/zinc­(II)­oxide (ZnO)/BHJ active layer/molybdenum trioxide (MoO_3_)/silver (Ag). The inverted OSC device architecture is illustrated in Figure 4a. In this study, PTB7-Th is chosen as the donor material for the S-NPN-EH- and POR6−4FIC-based active layers, owing to its favorable alignment of energy levels and complementary absorptions. Similarly, PBDB-T is chosen as the donor material for the T-NPN-OD-based active layer, due to its suitable energy-level alignment as well as complementary absorption. The detailed device optimization conditions are presented in Tables S1–S3. The detailed current density−voltage (*J−V*) curves of all optimized devices are demonstrated in Figure 4b, and the data are presented in Table 2. Under the standard AM1.5G illumination at 100 mW cm^−2^ condition (1 sun), the optimized device based on PTB7-Th/S-NPN-EH blend achieves a higher PCE of 2.19% with a *V*_oc_ of 0.80 V, a *J*_sc_ of 5.69 mA cm^−2^, and an FF of 48.15%, whereas the device based on the PBDB-T/T-NPN-OD blend shows a lower PCE of 1.01% with a *V*_oc_ of 0.74 V, a *J*_sc_ of 3.22 mA cm^−2^, and an FF of 42.53%. Due to the up-shifted LUMO of S-NPN-EH, the S-NPN-EH-based device exhibits a much higher *V*_oc_ compared with the T-NPN-OD-based device. Moreover, strong intra/intermolecular interactions of T-NPN-OD enhance the molecular aggregation, which increases crystallinity and domain size and thus decreases the exciton dissociation and charge transport. The reference PTB7-Th:POR6−4FIC-based device delivers a poor PCE of 0.32% with a *V*_oc_ of 0.70 V, a *J*_sc_ of 1.45 mA cm^−2^, and an FF of 31.62%. These results reveal that balanced active layer film morphology, well-matched energy levels, and appropriate solubility are more critical compared to the strong absorption profiles.

**Figure 4:**
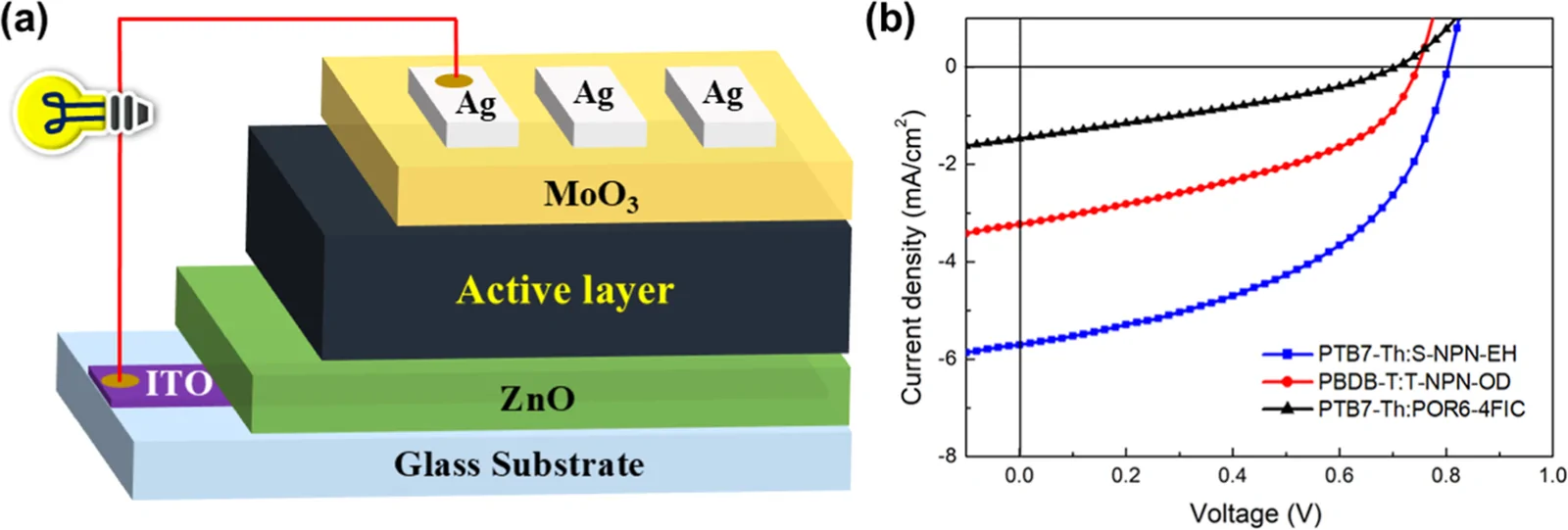
(a) Schematic illustration of an inverted BHJ OSC device architecture. (b) *J−V* characteristics of S-NPN-EH, T-NPN-OD, and POR6−4FIC OSC devices.

**Table 2:** Optimized Photovoltaic Performances of S-NPN-EH, T-NPN-OD, and POR6-4FIC Devices under the Illumination of AM1.5G (100 mW cm^−2^) Conditions

blend	*V*_oc_ (V)	*J*_sc_ (mA cm^−2^)	FF (%)	PCE (%)
PTB7-Th/S-NPN-EH	0.80	5.69	48.15	2.19 (2.12 ± 0.10)^a^
PBDB-T/T-NPN-OD	0.74	3.22	42.53	1.01 (0.96 ± 0.12)^a^
PTB7-Th/POR6−4FIC	0.70	1.45	31.62	0.32 (0.30 ± 0.08)^a^

a Average PCE data obtained over 8 individual device cells.

## 4 Conclusion

In summary, we successfully synthesized two porphyrin-based NFAs, called S-NPN-EH and T-NPN-OD, with porphyrin as the central core and NTI as terminal units linked with and without an acetylene π-bridge, respectively. For comparison to these molecules, we further synthesized POR6−4FIC, where the central porphyrin core is connected to two 2FIC as the terminal acceptor units linked with two acetylene π-bridged alkylthiophenes as the π-spacer. As a result, T-NPN-OD shows red-shifted absorption in the UV–vis–NIR region with intensified Soret-/Q absorption bands and down-shifted LUMO energy levels. In contrast, S-NPN-EH shows a pronounced and enhanced Soret absorption band in the UV–vis region and weaker Q-band absorption in the NIR region. In addition, S-NPN-EH elevates LUMO energy levels, resulting in enhanced *V*_oc_ values. The reference POR6−4FIC displays strong red-shifted absorption as well as down-shifted LUMO energy levels. As a consequence, the optimized device based on the PTB7-Th/S-NPN-EH blend achieves a higher PCE of 2.19% with a *V*_oc_ of 0.80 V, a *J*_sc_ of 5.69 mA cm^−2^, and an FF of 48.15% when compared with optimized PBDB-T:T-NPN-OD (PCE of 1.01%) and PTB7-Th/POR6−4FIC (PCE of 0.32%) devices. The improved photovoltaic performance of S-NPN-EH could be attributed to the fine-balanced blend film morphology, well-matched energy levels, and solubility compared to the strong absorption profiles of T-NPN-OD and POR6−4FIC. Overall, these results can help to further functionalize porphyrins to obtain high-efficiency OSCs containing porphyrins. While the OPV performance is not ideal, the molecular design and light-harvesting properties of porphyrin-based derivatives described herein hold potential in other applications, such as understanding energy transfer characteristics, light-to-fuel conversion, and photodynamic therapy, and may also provide insights into the future development of porphyrin-based light absorbers.

## Supplementary Material



## Notes

Synthetic details, spectral data of all the compounds (^1^H NMR, ^13^C NMR, and mass spectra), TGA/DSC thermograms, CV plots, DFT calculations, and BHJ-OSCs device fabrication (PDF)
