# Supplementary material for: 2-(5,6-Difluoro-3-oxo-2,3-dihydro-1H-inden-1-ylidene)-malononitrile and Naphtho[2,3-b]thiophene Diimide End-Capped Porphyrin Derivatives as Panchromatic Light-Harvesting Absorbers and Non-Fullerene Acceptors in Organic Solar Cells
Source: ACS Omega. 2026 Aug 31;11(36):54612–21. doi: 10.1021/acsomega.6c07199 (PMC13584800; doi:10.1021/acsomega.6c07199)
Supplement: Supplementary file 1 [file ao6c07199_si_001.pdf]

Supporting Information

2-(5,6-Difluoro-3-oxo-2,3-dihydro-1H-inden-1-ylidene)-malononitrile and Naphtho[2,3-*b*]thiophene Diimide End-Capped Porphyrin Derivatives as Panchromatic Light-Harvesting Absorbers and Non-Fullerene Acceptors in Organic Solar Cells

Manohar Reddy Busireddy,♀,a,b Yi-Chieh Chiu,♀,a,b Hung-Cheng Lin, a,b Chain-Shu Hsu*,a,b and Chia-Chih Chang*,a,b

[a]*Department of Applied Chemistry, National Yang Ming Chiao Tung University, 1001 University Road, Hsinchu 300093, Taiwan*

[b]*Center for Emergent Functional Matter Science, National Yang Ming Chiao Tung University, 1001 University Road, Hsinchu 300093, Taiwan*

E-mail: cshsu@nycu.edu.tw (Chain-Shu Hsu), cchang113ac@nycu.edu.tw (Chia-Chih Chang)

Note: ♀ = Both authors contributed equally to this work.

S.No.:	TABLE OF CONTENTS	Page No.
1.	Synthesis and Spectral Data of All Compounds	S-2
2.	Thermal Properties	S-25
3.	Cyclic Voltametric Properties	S-25
4.	DFT Calculations	S-26
5.	BHJ-OSCs Device Characteristics	S-27

1. Synthesis and Spectral Data of All Compounds

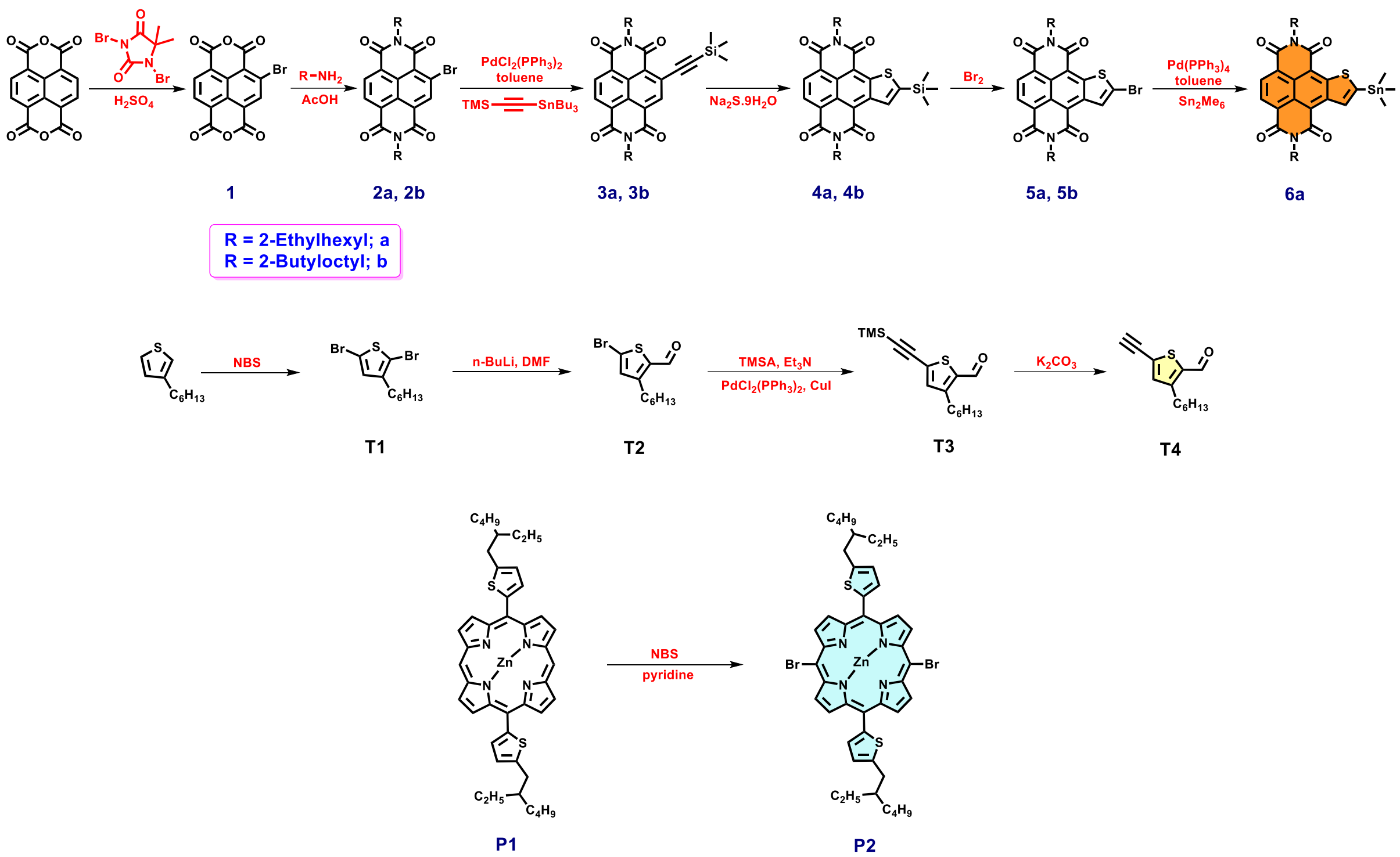

Scheme S1. Synthetic route of all monomers.

Synthesis of Compound 1. A solution of 1,3-dibromo-5,5-dimethylhydantoin (3.14g, 11 mmol) in sulphuric acid (30 mL) was added at room temperature (rt) to a 30 mL sulphuric acid solution of 1,4,5,8-naphthalenetetracarboxylic dianhydride (5.36 g, 20 mmol) over a period of 1 h. The resulting reaction mixture was stirred at rt for 12 h and then carefully poured onto ice to give a bright yellow precipitate. The solid was collected by filtration, washed with water and methanol, and dried under vacuum. The dried crude product (5.84 g) was used for the next reaction without further purification.

Synthesis of Compound 2a. The crude product 1 (3.00 g, 8.64 mmol) and 2-ethylhexylamine (2.63 g, 20.31 mmol) were added to 80 mL of acetic acid and stirred for 3 h at 120 °C. The

resulting reaction mixture was cooled to rt and poured into cold water to get a precipitate. The precipitate was filtered and washed with methanol. The crude precipitate was purified via silica gel column chromatography using dichloromethane (DCM):hexane (2:1) as eluent, followed by crystallization from DCM/MeOH to give compound 2a as a pale-yellow solid (1.65 g, yield = 33%). ^{1}H NMR (400 MHz, $CDCl_3$, ppm): δ 8.94 (s, 1H), 8.82 (d, *J* = 7.5 Hz, 1H), 8.76 (, *J* = 7.4 Hz, 1H), 4.15 (m, 4H), 1.94 (m, 2H), 1.41-1.25 (m, 16H), 0.91 (m, 12H).

Synthesis of Compound 2b. The crude product 1 (3.84 g, 11.06 mmol) and 2-octyldodecylamine (7.74 g, 26.01 mmol) were dissolved in 100 mL of acetic acid and stirred for 6 h at 120 °C. The reaction mixture was cooled to rt, quenched with water, and extracted with DCM. Then, the organic solution was washed with 10% KOH solution, water, and dried over anhydrous Na_2SO_4. After the evaporation of the solvent, the obtained crude product was purified by silica gel column chromatography using DCM:hexane (1:2) as an eluent, to give a colorless oil (2b, 2.34 g, yield = 23%). ^{1}H NMR (400 MHz, $CDCl_3$, ppm): δ 8.94 (s, 1H), 8.81 (d, *J* = 7.6 Hz, 1H), 8.78 (, *J* = 7.7 Hz, 1H), 4.13 (m, 4H), 1.98 (m, 2H), 1.43-1.18 (m, 64H), 0.87 (m, 12H).

Synthesis of Compound 3a. To a stirred solution of compound 2a (0.5 g, 0.88 mmol) in toluene (20 mL), $PdCl_2(PPh_3)_2$ (62 mg, 0.09 mmol) was added, and the resulting solution was further stirred for 30 min at rt under a nitrogen (N_2) atmosphere. Then, tributyl(trimethylsilylethynyl)tin (1.02 g, 2.63 mmol) was added, and the reaction mixture was refluxed for 1 h. The reaction mixture was cooled to rt, and the toluene was evaporated under vacuum. The resulting residue was purified by silica gel column chromatography using DCM:hexane (2:1) as an eluent, followed by recrystallization from DCM:MeOH to give a pale-yellow solid (3a, 0.45 g, yield = 88%). ^{1}HNMR (400 MHz, $CDCl_3$, ppm): δ 8.77 (s, overlapped,

1H), 8.76 (d, overlapped, *J* = 7.0 Hz, 1H), 8.71 (d, J = 7.7 Hz, 1H), 4.13 (m, 4H), 1.92 (m, 2H), 1.41-1.27 (m, 16H), 0.91 (m, 12H), 0.37 (s, 9H).

Synthesis of Compound 3a. A similar procedure to the synthesis of compound 3a with compound 2b afforded compound 3b as a colorless oil (yield = 93%). ^{1}H NMR (400 MHz, $CDCl_3$, ppm): δ 8.78 (s, overlapped, 1H), 8.77 (d, overlapped, *J* = 6.5 Hz, 1H), 8.72 (d, *J* = 7.7 Hz, 1H), 4.13 (m, 4H), 1.98 (m, 2H), 1.35-1.15 (m, 64H), 0.87 (m, 12H), 0.37 (s, 9H).

Synthesis of Compound 4a. Under the N_2 condition, sodium sulfide hydrate ($Na_2S{\cdot}9H_2O$, 4.12g, 17.15 mmol) was added to a stirred suspension of 3a (1.68 g, 2.86 mmol) in 2-methoxyrthanol (200 mL) and acetic acid (4.0 mL) at 60 °C. The resulting mixture was stirred at the same temperature for 12 h, then the mixture was cooled to rt and stirred for 3 h at rt under atmospheric conditions. Then, the reaction mixture was dropped into water to precipitate a red solid. The precipitate was collected by filtration and washed with water and methanol. The residue was purified by silica gel column chromatography using DCM:hexane (2:1) as an eluent, to give a red solid (4a, 1.09 g, yield = 61%). ^{1}H NMR (400 MHz, $CDCl_3$, ppm): δ 9.08 (s, 1H), 8.84 (s, 2H), 4.23 (m, 4H), 2.03 (m, 2H), 1.45-1.23 (m, 16H), 0.88 (m, 12H), 0.52 (s, 9H).

Synthesis of Compound 4b. Under the N_2 condition, sodium sulfide hydrate ($Na_2S{\cdot}9H_2O$, 3.12g, 13.0 mmol) was added to a stirred suspension of 3b (1.68 g, 2.86 mmol) in 2-methoxyrthanol (100 mL) and acetic acid (2.0 mL) at 60 °C. The resulting mixture was stirred at the same temperature for 12 h, then the mixture was cooled to rt and stirred for 3 h at rt under atmospheric conditions. Then, the reaction mixture was quenched with water, extracted with DCM, and

dried over Na_2SO_4. The resulting residue was purified by silica gel column chromatography using DCM:hexane (1:2) as eluent, to give a red solid (4b, 1.3 g, yield = 63%). %). ^{1}H NMR (400 MHz, $CDCl_3$, ppm): δ 9.08 (s, 1H), 8.84 (s, 2H), 4.22 (m, 4H), 2.07 (m, 2H), 1.35-1.15 (m, 64H), 0.86 (m, 12H), 0.52 (s, 9H).

Synthesis of Compound 5a. To a stirred solution of 4a (1.13 g, 1.83 mmol) in 100 mL of DCM, bromine (4.38 g, 27.4 mmol) was added at rt. After stirring at 40 °C for 15 h, the mixture was quenched with diluted $NaHSO_3$ aqueous solution and extracted with DCM. The combined organic layer was washed with saturated NaCl solution, dried over Na_2SO_4, and concentrated under reduced pressure. The resulting crude was purified by silica gel column chromatography using DCM:hexane (2:1) as an eluent, to give s orange solid (5a, 1.01g, yield = 87%). ^{1}H NMR (400 MHz, $CDCl_3$, ppm): δ 9.06 (s, 1H), 8.85 (s, 2H), 4.20 (m, 4H), 1.98 (m, 2H), 1.44-1.24 (m, 16H), 0.98-0.86 (m, 12H).

Synthesis of Compound 5b. A similar procedure to the synthesis of compound 5a with compound 4b afforded compound 5b as a yellow color oil (yield = 72%). ^{1}H NMR (400 MHz, $CDCl_3$, ppm): δ 9.03 (s, 1H), 8.83 (s, 2H), 4.18 (m, 4H), 2.02 (m, 2H), 1.35-1.15 (m, 64H), 0.86 (m, 12H).

Synthesis of Compound 6a. A solution of 5a (0.461 g, 0.74 mmol) and hexamethyldistannane (0.724 g, 2.2 mmol) in dry toluene (100 mL) was deoxygenated for 30 min. Tetrakis(triphenylphosphine)palladium(0) ($Pd(PPh_3)_4$, 0.085 g, 0.07 mmol) was added, and the reaction mixture was heated to 95 °C for 12 h. After cooling to rt, the solvent was evaporated under a rotary vacuum. The resulting residue was purified by silica gel column chromatography

using DCM:hexane (2:1) as an eluent, followed by recrystallization from DCM:MeOH to give an orange solid (6a, 0.448 g, yield = 86%). ^{1}H NMR (400 MHz, $CDCl_3$, ppm): δ 9.07 (s, 1H), 8.84 (s, 2H), 4.25 (m, 4H), 2.03 (m, 2H), 1.44-1.24 (m, 16H), 0.92 (m, 12H), 0.56 (s, 9H).

Synthesis of Compound T1. 3-Hexylthiophene (4.8 g, 28.52 mmol) was dissolved in 200 mL of $CHCl_3$ and then cooled to 0 °C. At 0 °C, NBS (12.19 g, 68.49 mmol) was added portion-wise over 30 min, then the reaction mixture was stirred at rt for 12 h. The reaction mixture was quenched with water and extracted with DCM. The combined organic layer was dried over Na_2SO_4 and concentrated under vacuum. The resulting residue was purified by silica gel column chromatography using hexane as an eluent, to give compound T1 (8.5 g, yield = 91%). ^{1}H NMR (400 MHz, $CDCl_3$, ppm): δ 6.77 (s, 1H), 2.50 (t, 2H), 1.35-1.25 (m, 8H), 0.88 (t, 3H).

Synthesis of Compound T2. Compound T1 (8.45 g, 25.91 mmol) was dissolved in dry THF (200 mL), then *n*-butyllithium (10.4 mL, 2.5 M in hexane, 25.85 mmol) was added at -78 °C under N_2 protection and stirred for 1 h at the same temperature. Then, anhydrous DMF was added to the reaction mixture at -78 °C. After the addition, the solution was slowly warmed to rt and stirred for 12 h at rt. The reaction mixture was poured into 2N HCl, and the organic layer was extracted with hexane, dried over Na_2SO_4, and concentrated under vacuum pressure. The residue was purified by silica gel column chromatography using DCM:hexane (1:1) as an eluent, to give compound T2 (4.15 g, yield = 58%). ^{1}H NMR (400 MHz, $CDCl_3$, ppm): δ 9.75 (s, 1H), 7.45 (s, 1H), 2.59 (t, 2H), 1.35-1.25 (m, 8H), 0.89 (t, 3H).

Synthesis of Compound T3. In a two-necked 50 mL round-bottom flask, compound T2 (185 mg, 0.67 mmol), $Pd(PPh_3)Cl_2$ (47 mg, 0.07 mmol), CuI (13 mg, 0.07 mmol) were mixed in

THF (20 mL) and triethylamine (20 mL). Then, trimethylsilylacetylene (198 mg, 2.02 mmol) was added to the reaction mixture and stirred for 12 h at 50 °C. After the completion of the reaction, the solvent was removed by rotary vacuum to obtain the crude product. The crude product was redissolved in DCM and washed with an aqueous NH_4Cl solution, and the organic layer was dried over Na_2SO_4 and concentrated. The residue was purified by silica gel column chromatography using DCM:hexane (1:2) as an eluent, to give compound T3 (178 mg, yield = 91%). ^1H NMR (400 MHz, $CDCl_3$, ppm): δ 9.80 (s, 1H), 7.50 (s, 1H), 2.71 (t, 2H), 1.36-1.24 (m, 8H), 0.89 (t, 3H), 0.27 (s, 9H).

Synthesis of Compound T4. Compound T3 (178 mg, 0.61 mmol), K_2CO_3 (8.4 mg, 0.06 mmol), and 30 mL methanol were mixed in a 250 mL flask and stirred for 1 h at rt. Then, the organic layer was extracted with DCM, dried over Na_2SO_4, and concentrated under reduced pressure. The resulting crude product was purified by column chromatography on silica gel using DCM as the eluent, affording compound T4 (90 mg, yield = 67%). ^1H NMR (400 MHz, $CDCl_3$, ppm): δ 9.82 (s, 1H), 7.53 (s, 1H), 2.71 (t, 2H), 1.36-1.26 (m, 8H), 0.89 (t, 3H).

Synthesis of Compound P2. Compound P1 (0.1 g, 0.13 mmol) and pyridine (8mL) were dissolved in 100 mL of $CHCl_3$ and cooled to 0 °C. At 0 °C, *N*-bromosuccinimide (0.049 g, 0.28 mmol) was added and stirred for 30 min at the same temperature. Then, the reaction mixture was quenched with acetone, and the solvent was removed under reduced pressure. The resulting residue was purified by column chromatography on silica gel using DCM:hexane (1:3) as the eluent, followed by recrystallization from DCM:methanol to give a purple solid (P2, 0.1 g, yield = 83%). ^1H NMR (400 MHz, $CDCl_3$, ppm): δ 9.72 (d, J = 4.7 Hz, 4H), 9.22 (d, J = 4.7

Hz, 4H), 7.68 (d, *J* = 3.2 Hz, 2H), 7.17 (d, *J* = 3.2 Hz, 2H), 3.10 (d, *J* = 7.1 Hz, 4H), 1.86 (m, 2H), 1.45-1.20 (m, 16H), 1.15-0.98 (m, 12H).

Synthesis of Compound P3. To a stirred solution of compound P2 (113 mg, 0.12 mmol) in 20 mL of THF (20 mL), triethylamine (5 mL) was added. The resulting mixture was purged with N_2 for 30 min. Then, $Pd(PPh_3)_2Cl_2$ (7.6 mg, 0.01 mmol), CuI (2.1 mg, 0.01 mmol), and trimethylsilylacetalene (43 mg, 0.43 mmol) were added. The reaction mixture was refluxed for 12 h under N_2 conditions. The reaction solution was cooled to rt and quenched with a brine solution. The organic layer was extracted with DCM (3 times), dried over Na_2SO_4, and concentrated under vacuum pressure. Finally, the residue was purified by silica gel column chromatography using DCM:hexane (1:3) as an eluent, to give compound P3 (86 mg, yield = 73%). ^{1}H NMR (400 MHz, $CDCl_3$, ppm): δ 9.68 (d, *J* = 4.6 Hz, 4H), 9.18 (d, *J* = 4.6 Hz, 4H), 7.68 (d, *J* = 3.3 Hz, 2H), 7.16 (d, *J* = 3.3 Hz, 2H), 3.09 (d, *J* = 7.0 Hz, 4H), 1.87 (m, 2H), 1.68-1.44 (m, 16H), 1.125-0.98 (m, 12H), 0.61 (s, 18H).

Synthesis of Compound P4. Tetrabutylammonium fluoride (0.4 mL, 1M in THF, 0.04 mmol) was added to a stirred solution of compound P3 (159 mg, 0.17 mmol) in THF (20 mL). After the reaction mixture was stirred for 30 min, water was added to quench the reaction. Then, the compound was extracted with DCM, washed with water, dried over Na_2SO_3, and concentrated under vacuum pressure. The residue was purified by silica gel column chromatography using DCM:hexane (1:1) as an eluent, to give compound P4 (104 mg, yield = 77%). ^{1}H NMR (400 MHz, $CDCl_3$, ppm): δ 9.72 (d, *J* = 4.6 Hz, 4H), 9.22 (d, *J* = 4.6 Hz, 4H), 7.69 (d, *J* = 3.3 Hz, 2H), 7.17 (d, *J* = 3.3 Hz, 2H), 4.18 (s, 2H), 3.09 (d, *J* = 7.0 Hz, 4H), 1.87 (m, 2H), 1.68-1.44 (m, 16H), 1.11-0.98 (m, 12H).

Synthesis of Target Compound S-NPN-EH. In a dried 100 mL two-necked round-bottom flask, compound P2 (100 mg, 0.11 mmol) and compound 6a (232 mg, 0.33 mmol) were dissolved in 30 mL of anhydrous toluene, and the mixture was deoxygenated with N_2 for 30 min. Then, $Pd(PPh_3)_4$ was added to the reaction mixture and refluxed overnight under N_2 protection. The reaction mixture was cooled to rt and the solvent was removed by reduced pressure. The residue was purified by silica gel column chromatography using DCM:hexane (2:1) as an eluent, followed by recrystallization from DCM:methanol to give a purple solid (S-NPN-EH, 141 mg, yield = 70%). ^{1}H NMR (400 MHz, $CDCl_3$, ppm): δ 9.82 (s, 2H), 9.25 (m, 8H), 8.93 (m, 4H), 7.73 (d, J = 3.4 Hz, 2H), 7.15 (d, J = 3.4 Hz, 2H), 4.26 (m, 8H), 3.07 (d, J = 6.6 Hz, 4H), 2.04 (m, 4H), 1.83 (m, 2H), 1.45-1.24 (m, 48H). 1.07-1.01 (m, 12H), 0.99-0.81 (m, 24H); ^{13}C NMR (150 MHz, $CDCl_3$, ppm): δ 163.73, 163.50, 163.26, 163.21, 158.15, 151.76, 149.79, 147.35, 146.59, 143.00, 140.30, 133.55, 132.90, 131.69, 130.40, 130.17, 126.25, 125.65, 125.40, 124.34, 123.79, 118.75, 117.80, 114.90, 111.99, 44.68, 44.25, 41.69, 37.83, 37.79, 34.40, 32.65, 30.63, 30.58, 29.00, 28.53, 28.47, 25.92, 23.98, 23.93, 23.07, 23.05, 23.00, 14.18, 14.06, 14.02, 11.02, 10.58, 10.52; HRMS (FD) m/z calcd for $C_{108}H_{120}N_8O_8S_4Zn$: $[M]^+$ 1848.740, found = 1848.739.

Synthesis of Target Compound T-NPN-OD. In a dried 100 mL two-necked round-bottom flask, compound P4 (93 mg, 0.11 mmol), compound 5b (333 mg, 0.35 mmol), and diisopropylamine (10 mL) were dissolved in 20 mL of anhydrous THF, and the mixture was deoxygenated with N_2 for 30 min. Then, $Pd(PPh_3)_4$ and CuI (5 mg, 0.0 mmol) were added to the reaction mixture and stirred for 12 h at 50 °C under N_2 protection. After the completion of the reaction, the solvent was removed by a rotary vacuum to obtain the crude product. The crude was

redissolved in DCM and washed with an aqueous NH_4Cl solution, and the organic layer was dried over Na_2SO_4 and concentrated. The residue was purified by silica gel column chromatography using DCM:hexane (1:3 to 2:1) as an eluent, followed by recrystallization from DCM:methanol to give a black solid (T-NPN-OD, 148 mg, yield = 50%). ^{1}H NMR (600 MHz, d_6-THF, ppm): δ 9.81-8.95 (m, overlapped, 5H), 8.90-8.49 (m, overlapped, 5H), 8.48-8.19 (m, overlapped, 4H), 8.18-7.86 (m, overlapped, 2H), 7.54-7.46 (m, 2H), 4.42 (m, 8H), 3.33 (m, 4H), 2.17 (m, 6H), 1.92-1.10 (m, overlapped, 156H), 0.89-0.81 (m, 24H); ^{1}H NMR (150 MHz, d_6-THF, ppm): δ 164.17, 164.00, 163.46, 163.39, 151.51, 148.22, 141.47, 134.68, 133.20, 130.29, 126.93, 126.14, 125.48, 116.80, 106.43, 45.68, 42.82, 37.52, 37.44, 35.23, 33.64, 32.69, 31.07, 30.53, 30.23, 30.17, 29.96, 27.37, 26.70, 25.60, 24.04, 23.40, 14.63, 14.31, 11.36; MALDI-TOF: calcd for $C_{160}H_{216}N_8O_8S_4Zn$ $[M]^+$ 2569.491, found = 2571.946.

Synthesis of Compound P5. In a dried 100 mL two-necked round-bottom flask, compound P2 (360 mg, 0.39 mmol), compound T4 (369 mg, 1.67 mmol), and triethylamine (10 mL) were dissolved in 30 mL of anhydrous THF, and the mixture was deoxygenated with N_2 for 30 min. Then, $Pd(PPh_3)_4$ (60 mg, 0.05 mmol) and CuI (12 mg, 0.06 mmol) were added to the reaction mixture and stirred for 12 h at 50 °C under N_2 protection. After the completion of the reaction, the solvent was removed by a rotary vacuum to obtain the crude product. The crude was redissolved in DCM and washed with an aqueous NH_4Cl solution, and the organic layer was dried over Na_2SO_4 and concentrated. The residue was purified by silica gel column chromatography using THF:hexane (1:5) as an eluent, followed by recrystallization from DCM:methanol to give a green solid (P5, 382 mg, yield = 81%). ^{1}H NMR (400 MHz, $CDCl_3$, ppm): δ 9.79 (s, 2H), 9.21 (d, J = 4.6 Hz, 4H), 9.10 (d, J = 4.6 Hz, 4H), 7.74 (d, J = 3.3 Hz, 2H), 7.54 (s, 2H), 7.22 (d, J = 3.3 Hz, 2H), 3.13 (d, J = 6.7 Hz, 4H), 2.92 (t, 4H), 1.89 (m, 2H), 1.72-1.34 (m, 32H), 1.12 (t, 6H), 1.03 (t, 6H), 0.88 (t, 6H).

Synthesis of Target Compound POR6-4FIC. Under the N_2 protection, 2-(5,6-difluoro-3-oxo-2,3-dihydro-1*H*-inden-1-ylidene)malononitrile (90 mg, 0.39 mmol) and compound P5 (116 mg, 0.09 mmol) were dissolved in $CHCl_3$ (15 mL). The resulting mixture was deoxygenated with N_2 for 30 min, then a few drops of pyridine were added and stirred at 60 °C for 1 h under N_2 conditions. After the completion of the reaction, the reaction mixture was dropped into methanol to get a black precipitate. The precipitate was collected by filtration, washed with methanol and DCM, followed by recrystallization from THF/methanol to give a dark green solid (POR6-4FIC, 135 mg, yield = 86%). ^{1}H NMR (600 MHz, d_8-THF, ppm): δ 9.56 (d, *J* = 4.3 Hz, 4H), 9.19 (d, *J* = 4.5 Hz, 4H), 8.14 (m, 4H), 7.90 (d, *J* = 3.3 Hz, 2H), 7.36 (d, *J* = 3.0 Hz, 2H), 3.26 (d, 8H), 1.89 (m, 2H), 1.68-1.42 (m, 32H), 1.18 (t, 6H), 1.08 (t, 6H), 0.97 (m, 6H); ^{13}C NMR (150 MHz, d_8-THF, ppm): δ 157.16, 156.09, 153.77, 152.68, 152.01, 149.05, 148.04, 145.44, 141.22, 137.04, 136.45, 136.02, 134.46, 133.70, 131.03, 125.41, 123.04, 117.16, 114.58, 114.30, 112.52, 108.80, 101.88, 91.94, 42.78, 35.05, 34.65, 33.63, 32.64, 31.28, 30.67, 30.22, 29.93, 26.77, 25.59, 23.87, 23.47, 14.41, 14.35, 11.08; MALDI-TOF: calcd for $C_{94}H_{80}F_4N_8O_2S_4Zn$ $[M]^+$ 1620.451, found = 1622.013.

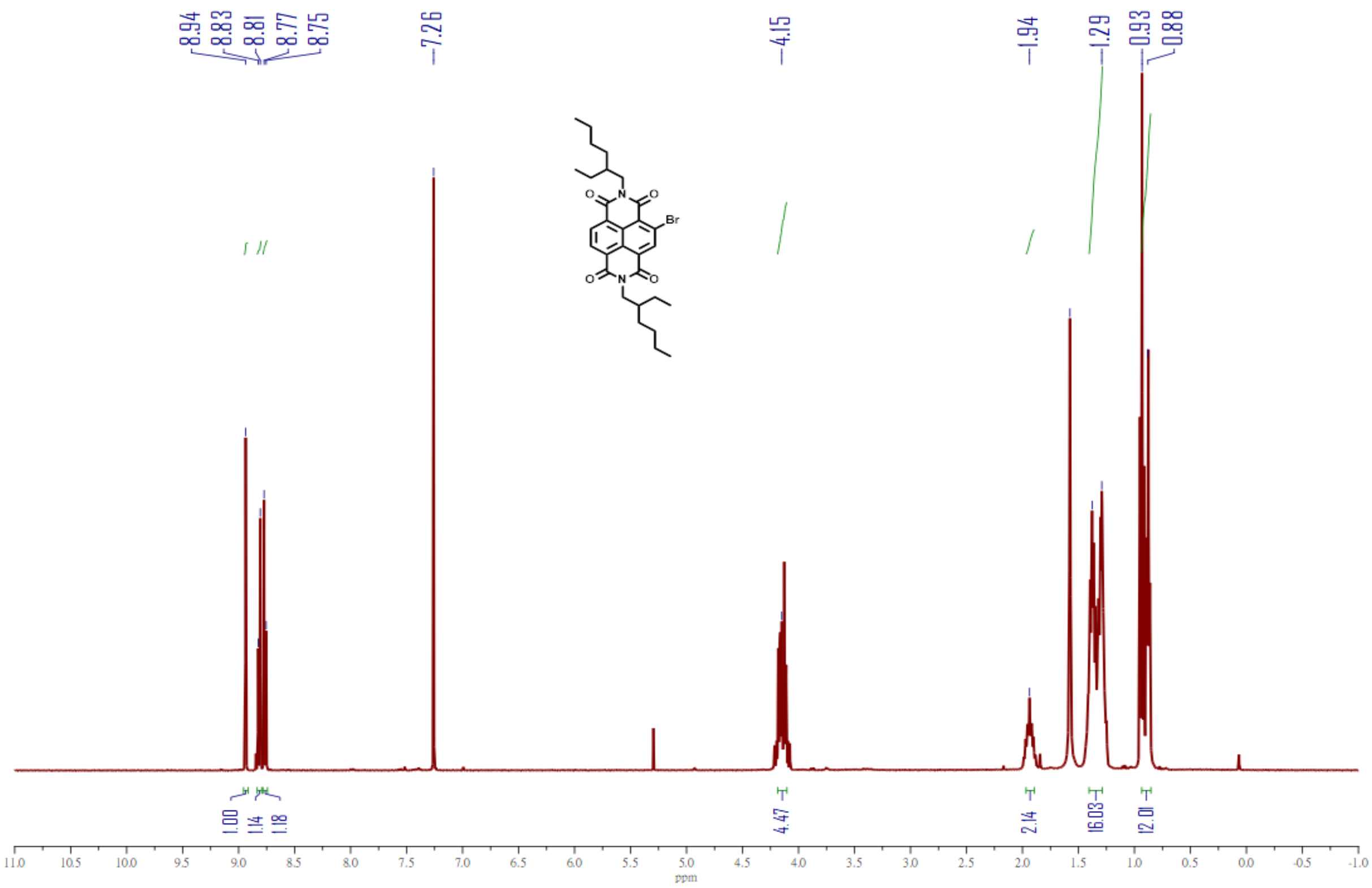

Figure S1. ^{1}H NMR spectrum of compound 2a.

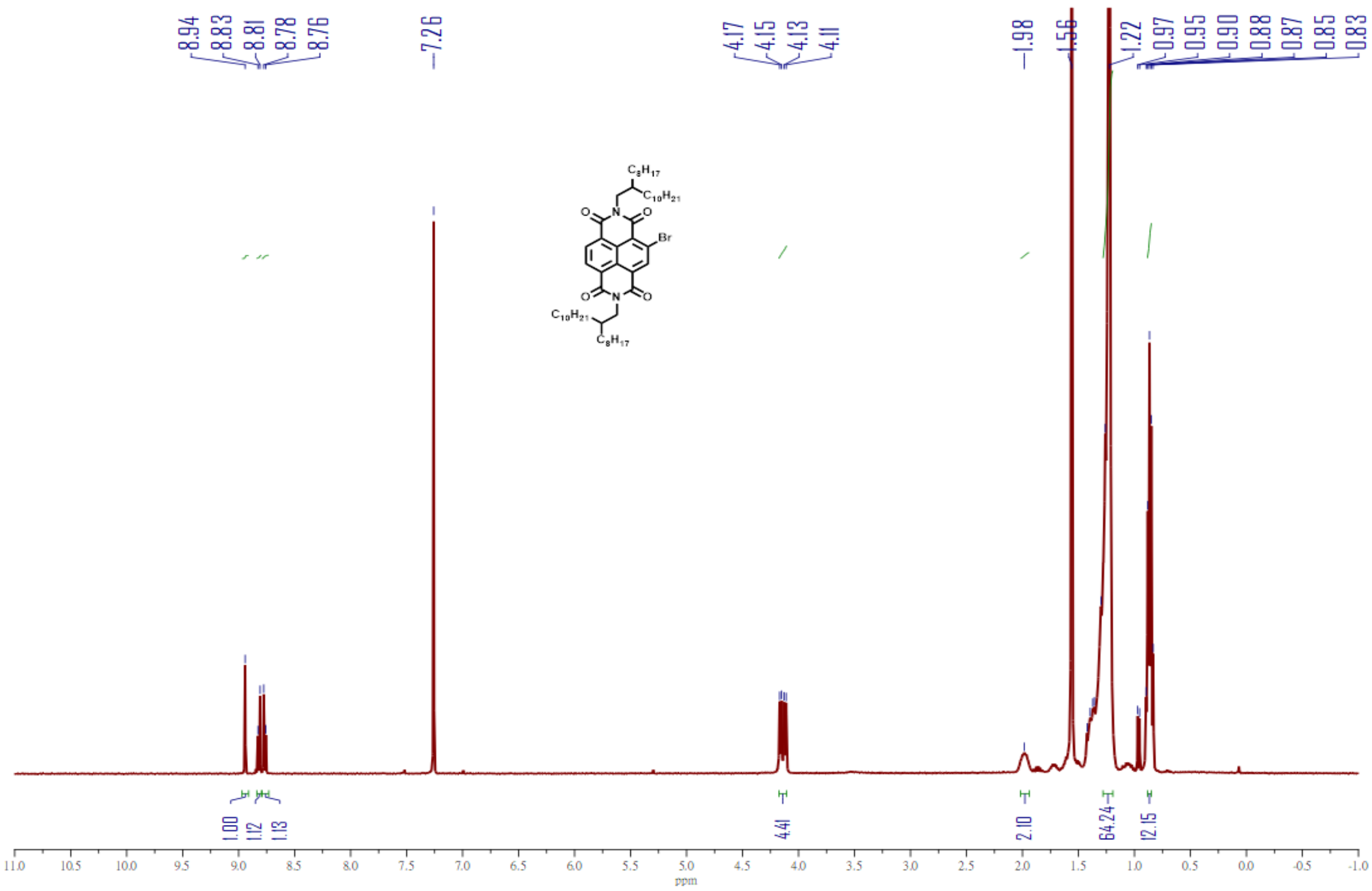

Figure S2. ^{1}H NMR spectrum of compound 2b.

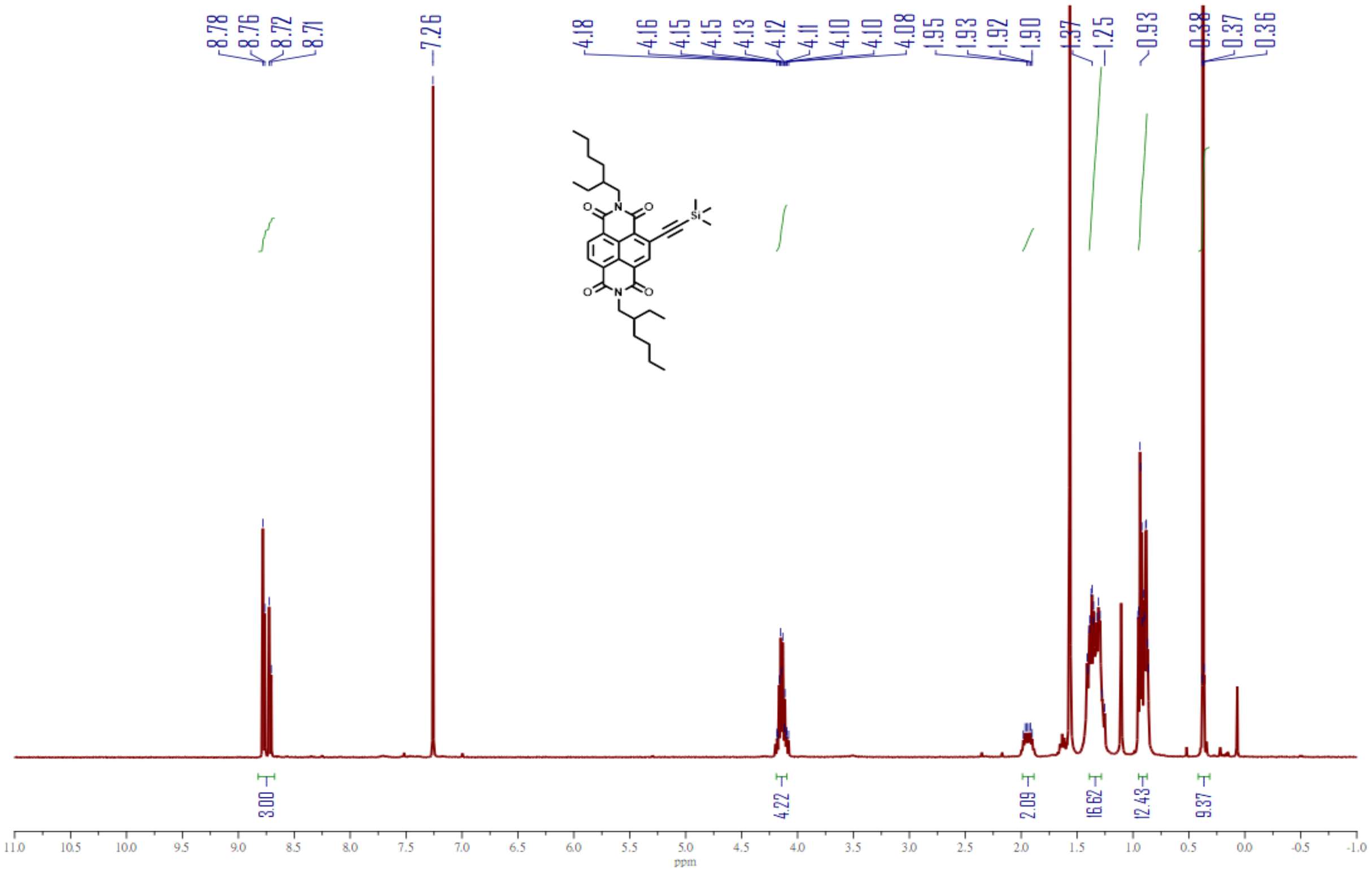

Figure S3. ^{1}H NMR spectrum of compound 3a.

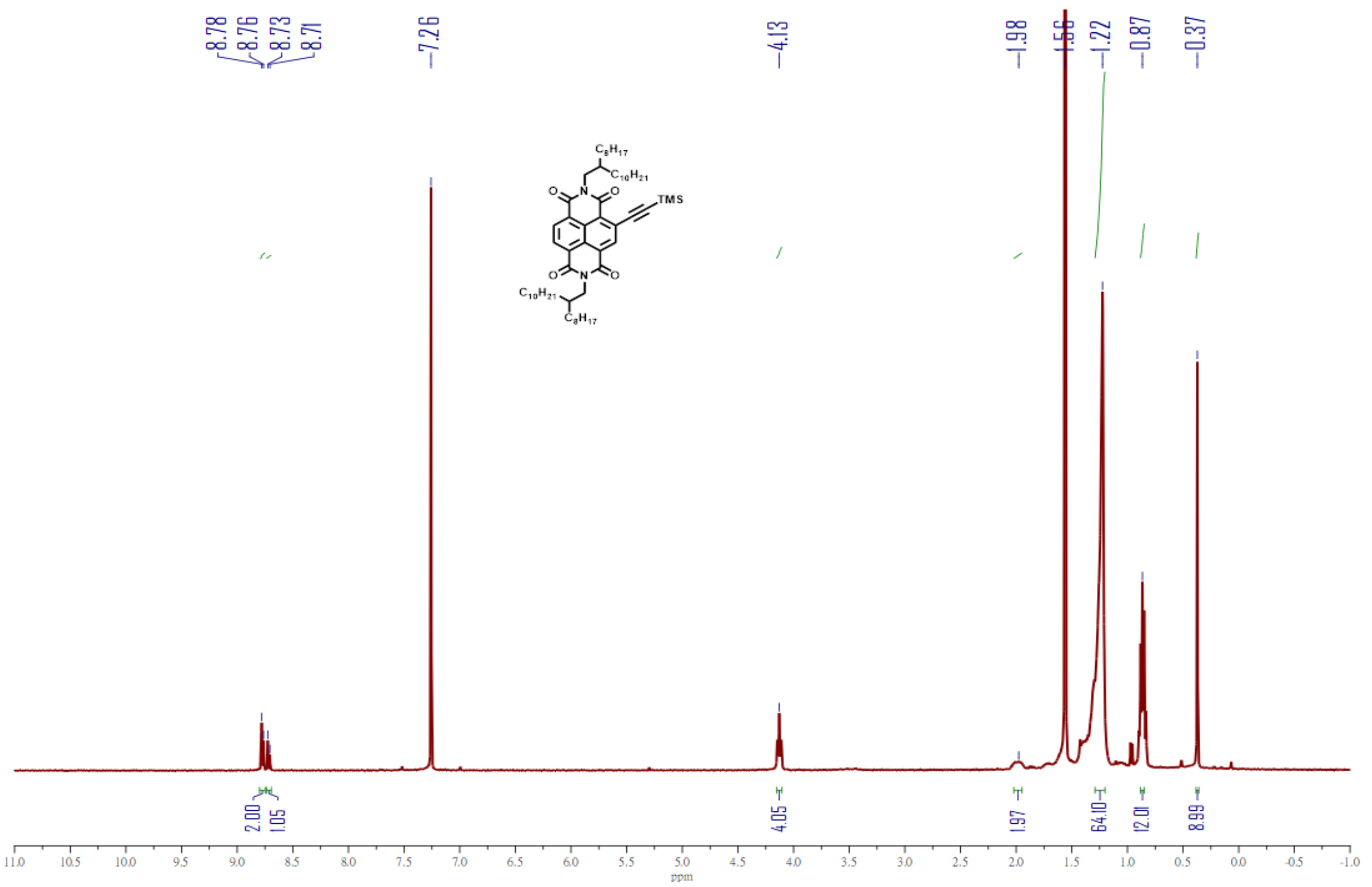

Figure S4. ^{1}H NMR spectrum of compound 3b.

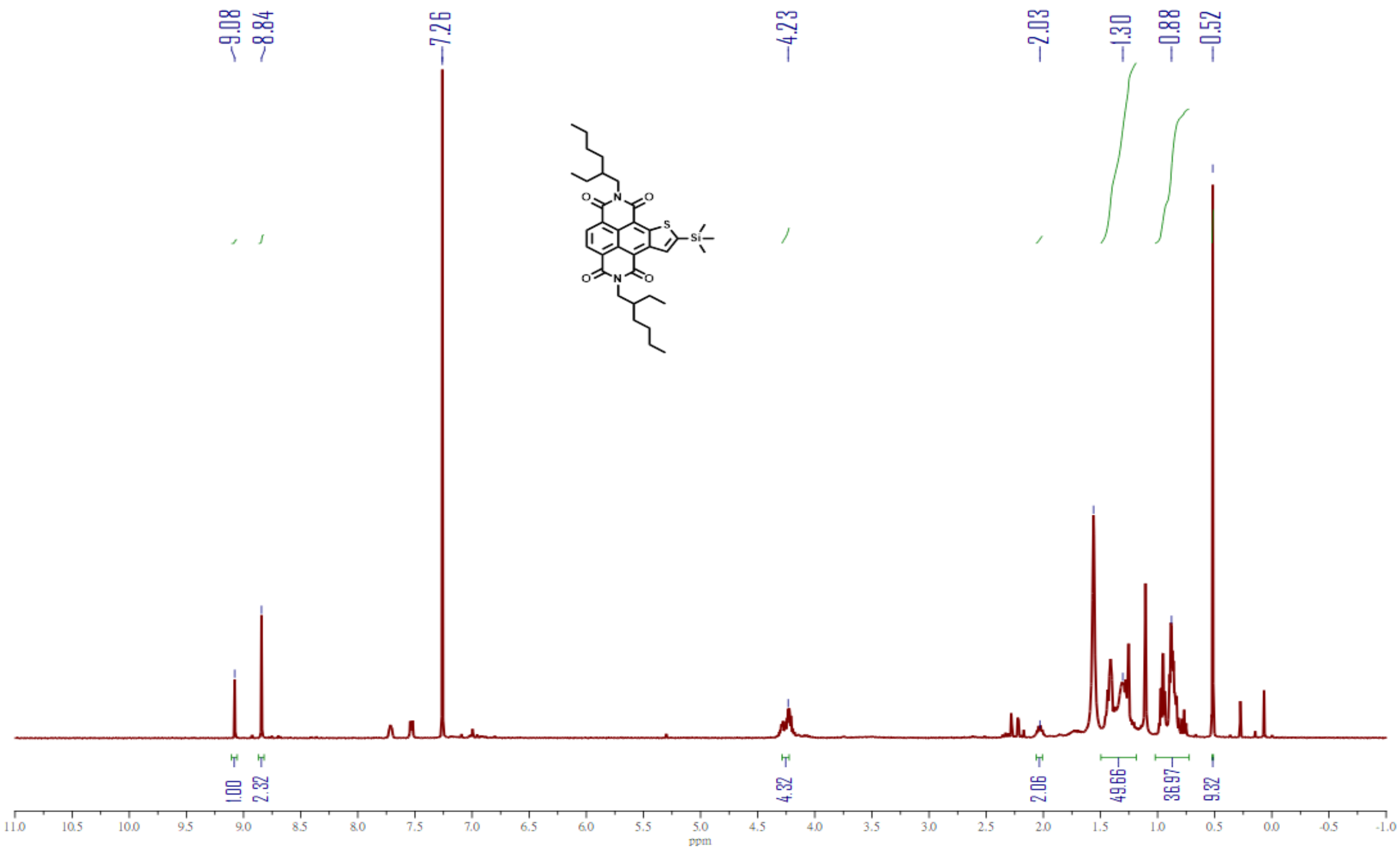

Figure S5. 1H NMR spectrum of compound 4a.

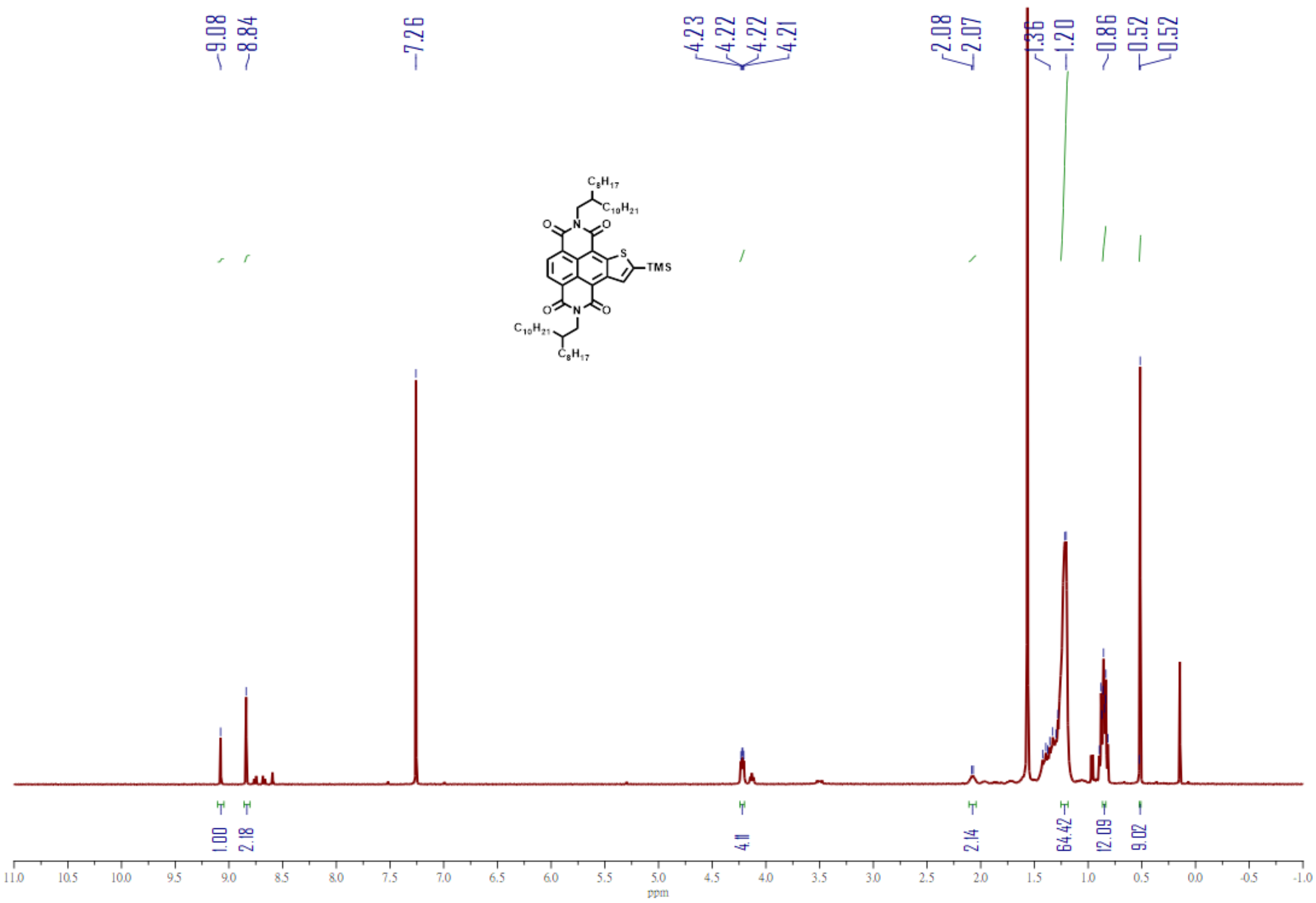

Figure S6. 1H NMR spectrum of compound 4b.

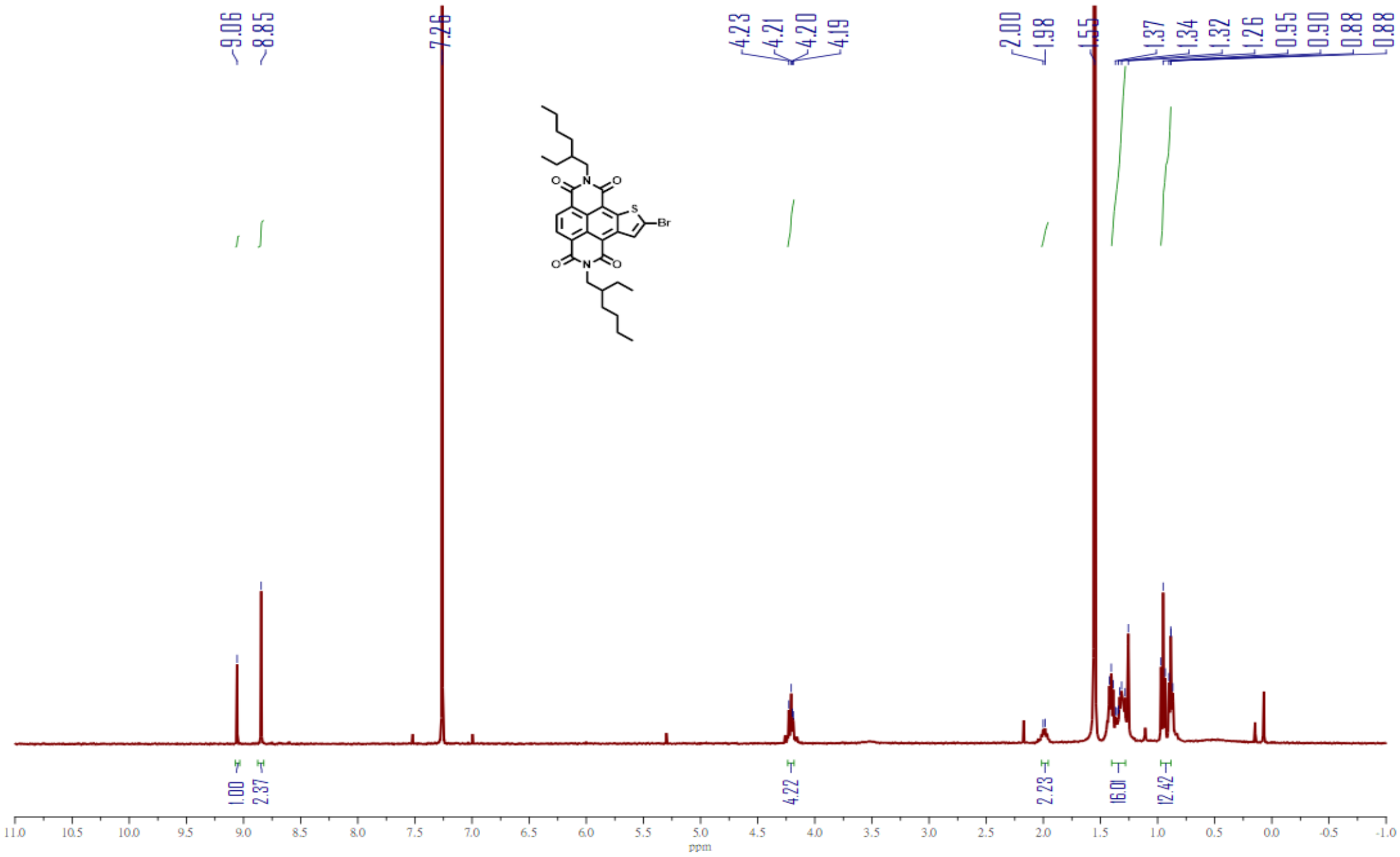

Figure S7. ^{1}H NMR spectrum of compound 5a.

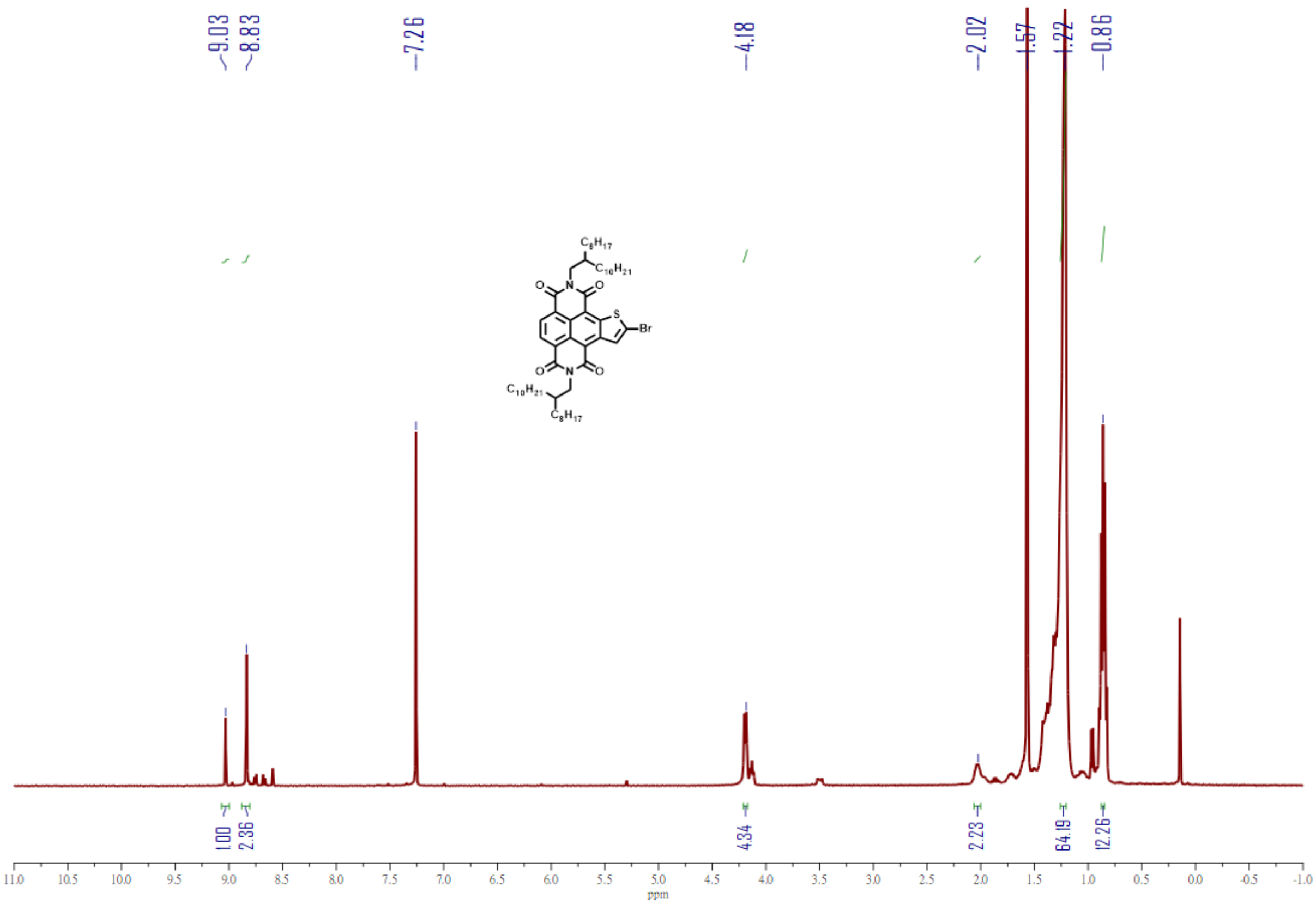

Figure S8. ^{1}H NMR spectrum of compound 5b.

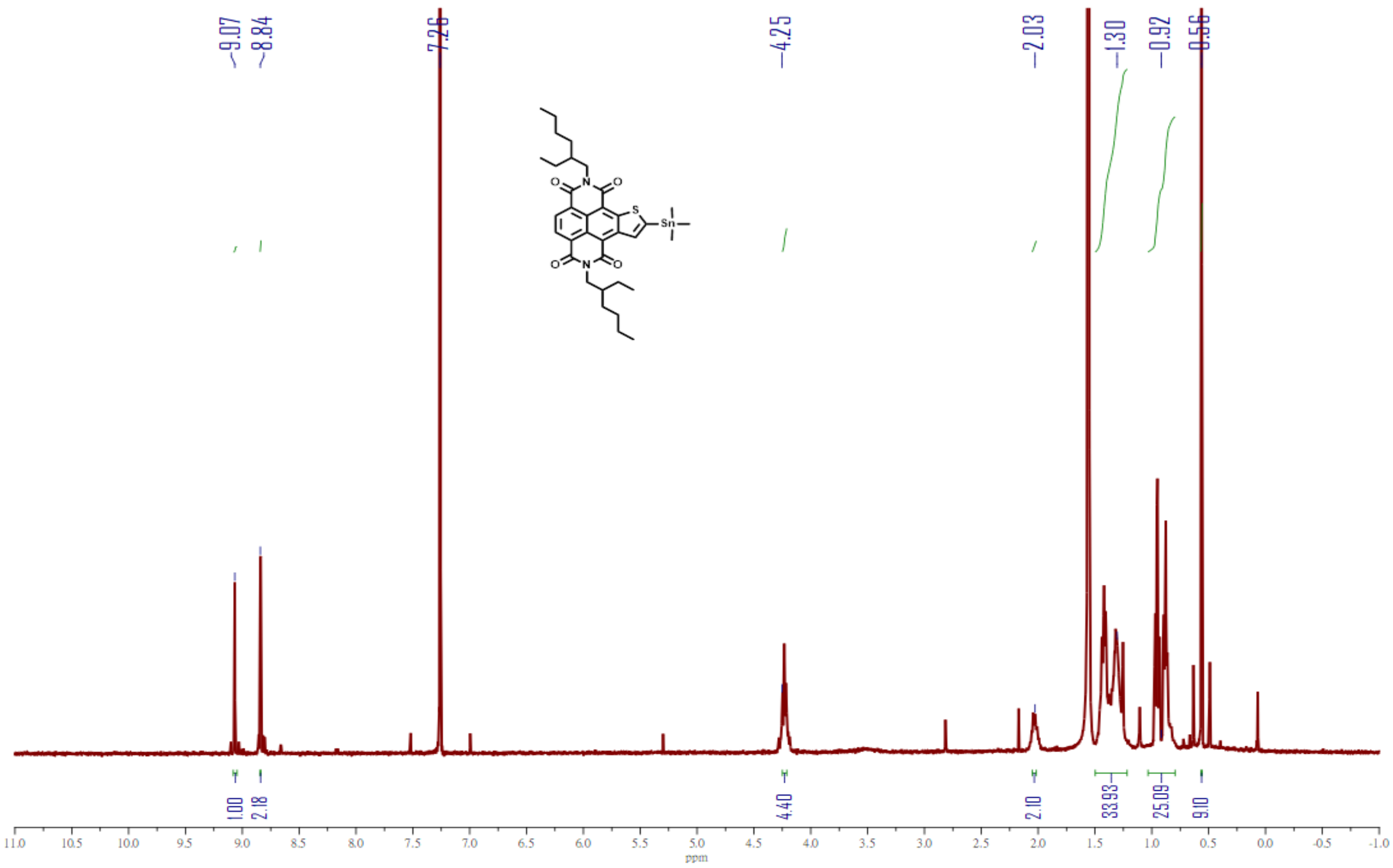

Figure S9. ^{1}H NMR spectrum of compound 6a.

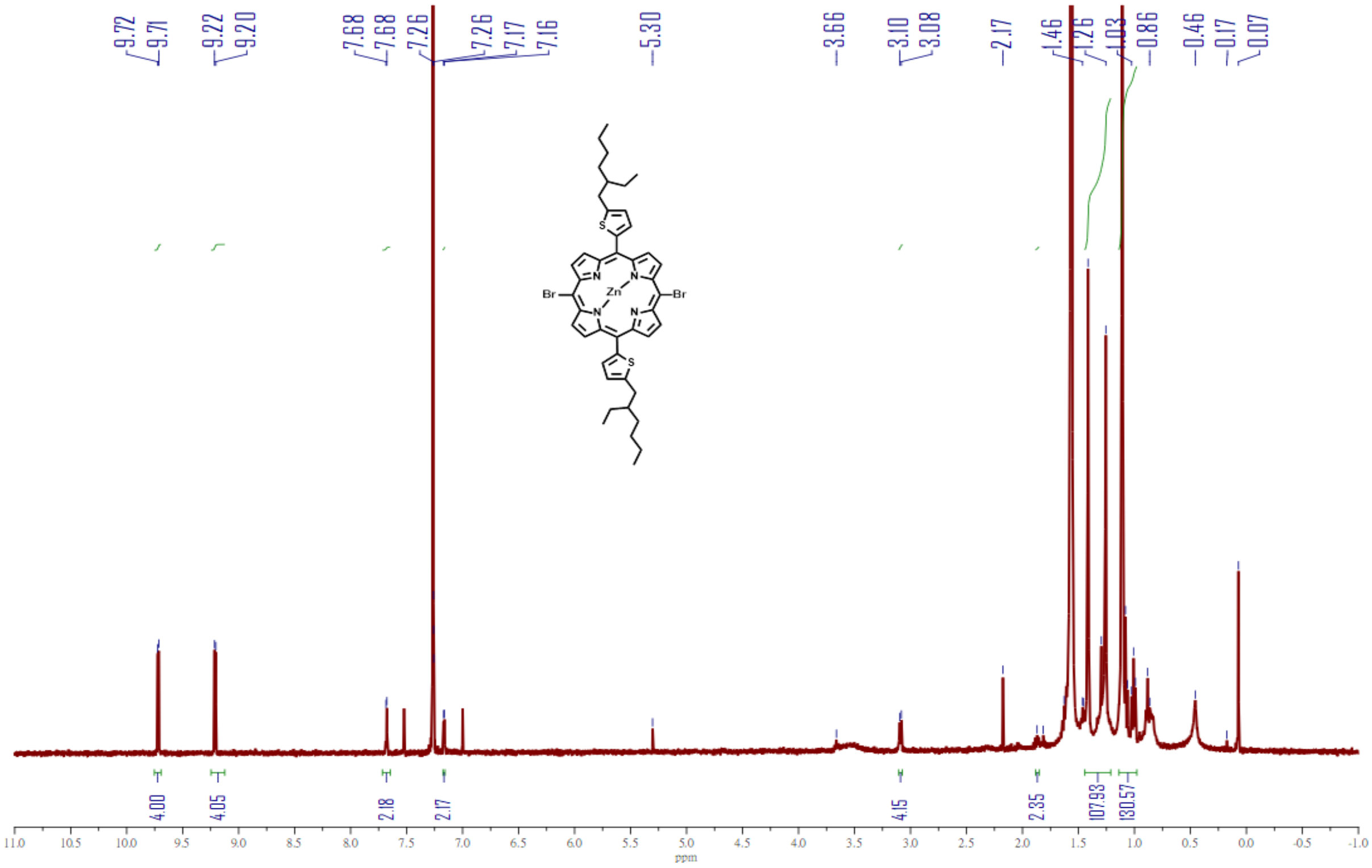

Figure S10. ^{1}H NMR spectrum of compound P2.

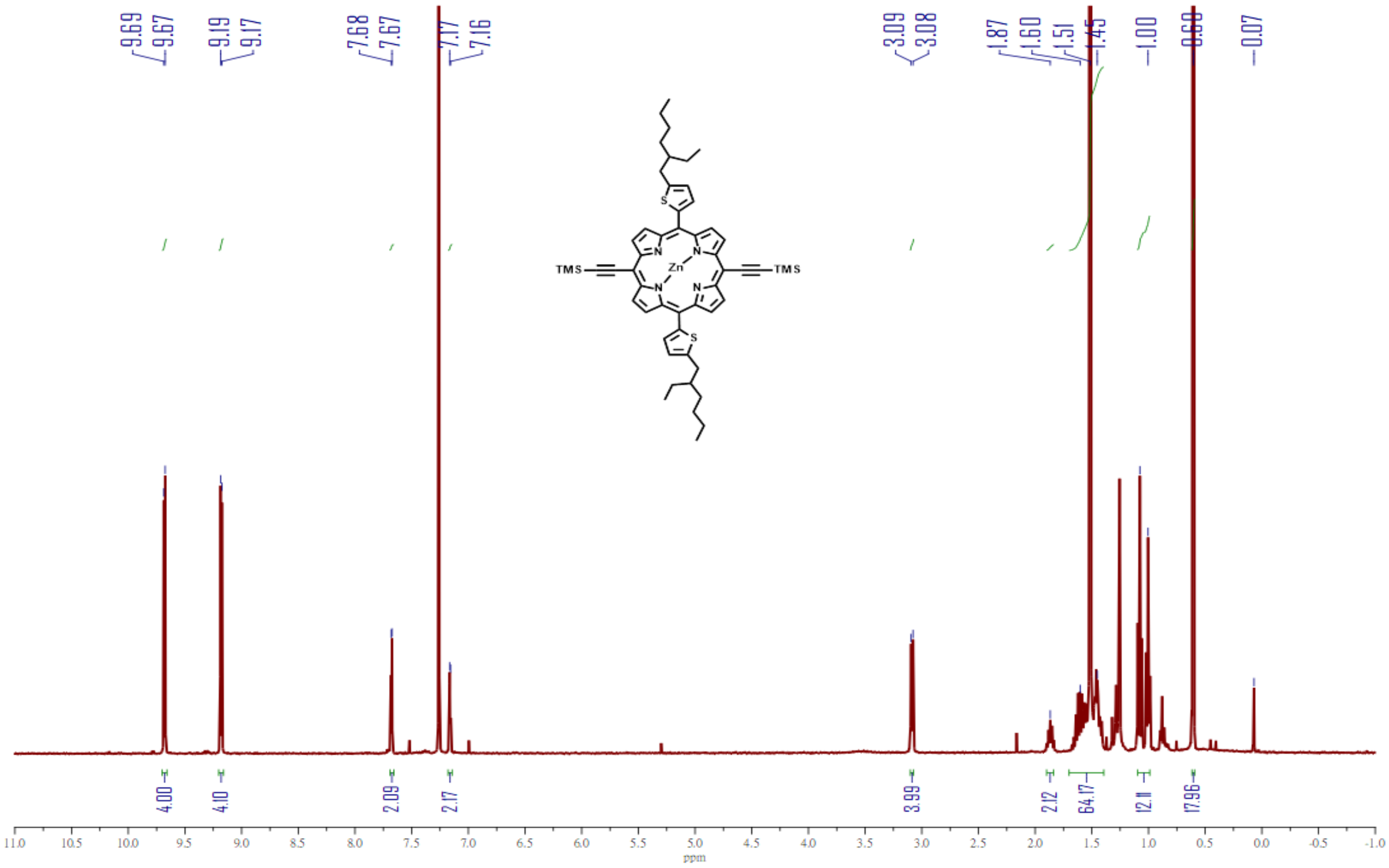

Figure S11. ^{1}H NMR spectrum of compound P3.

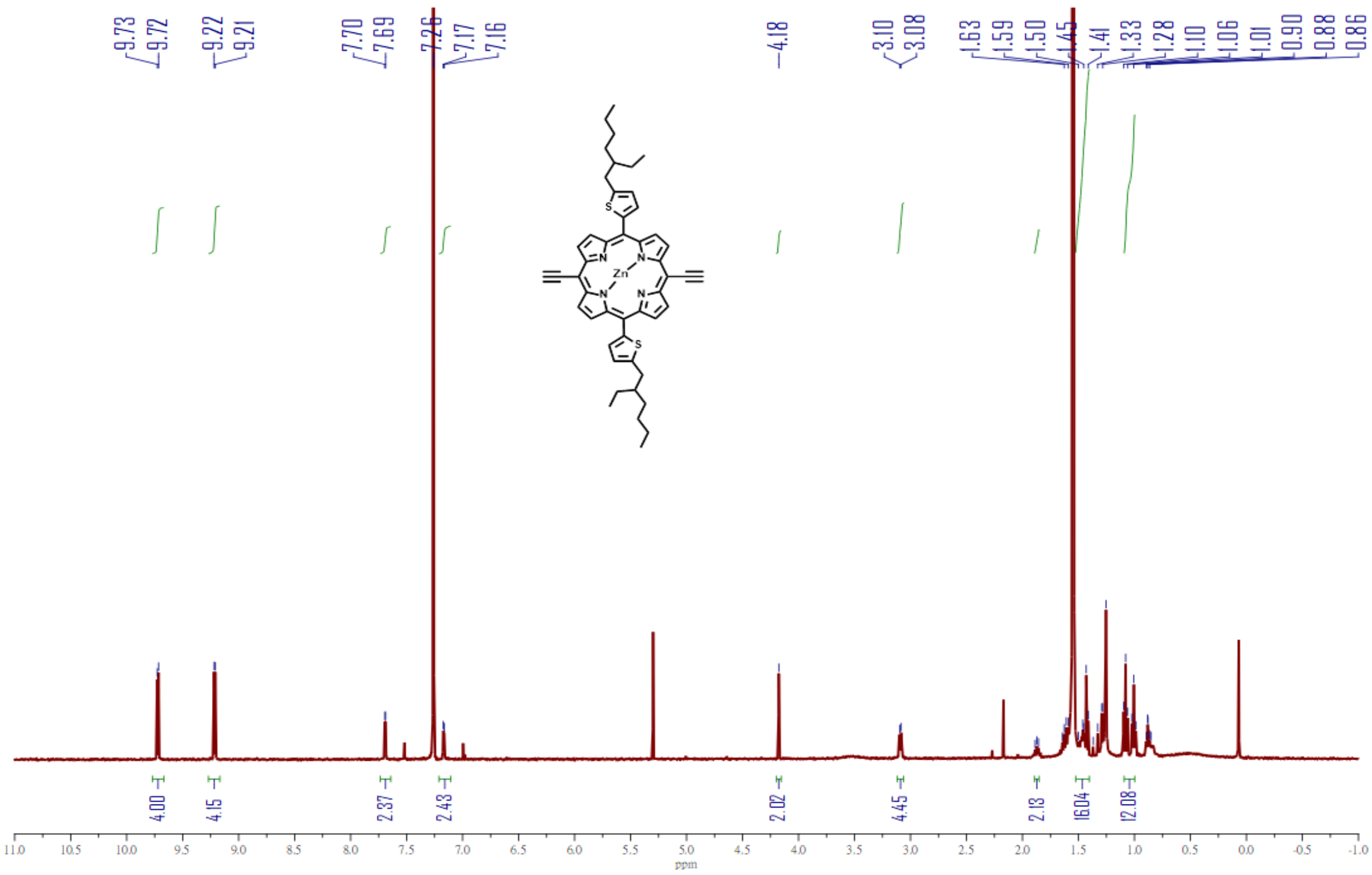

Figure S12. ^{1}H NMR spectrum of compound P4.

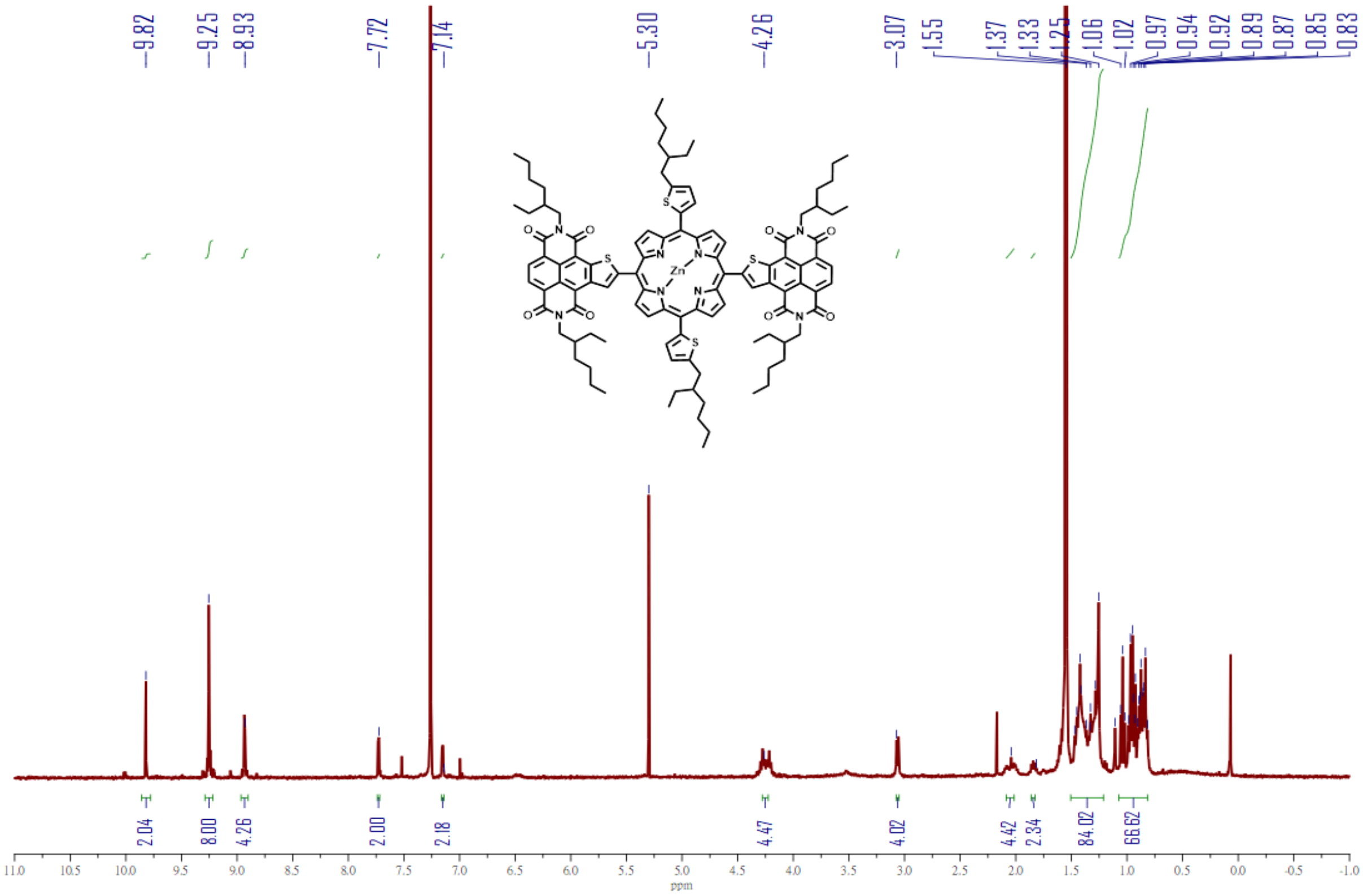

Figure S13. ¹H NMR spectrum of S-NPN-EH.

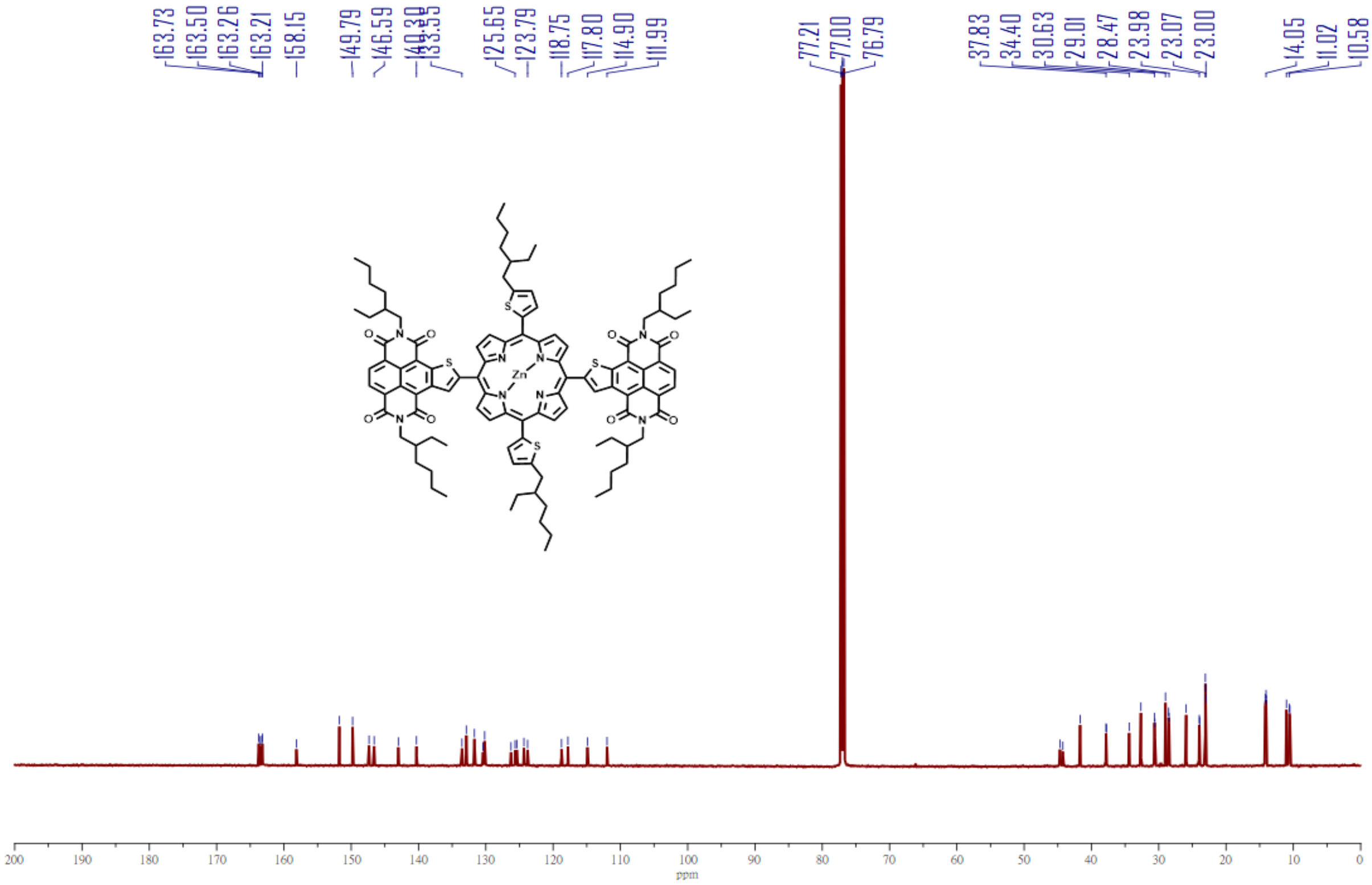

Figure S14. ¹³C NMR spectrum of S-NPN-EH.

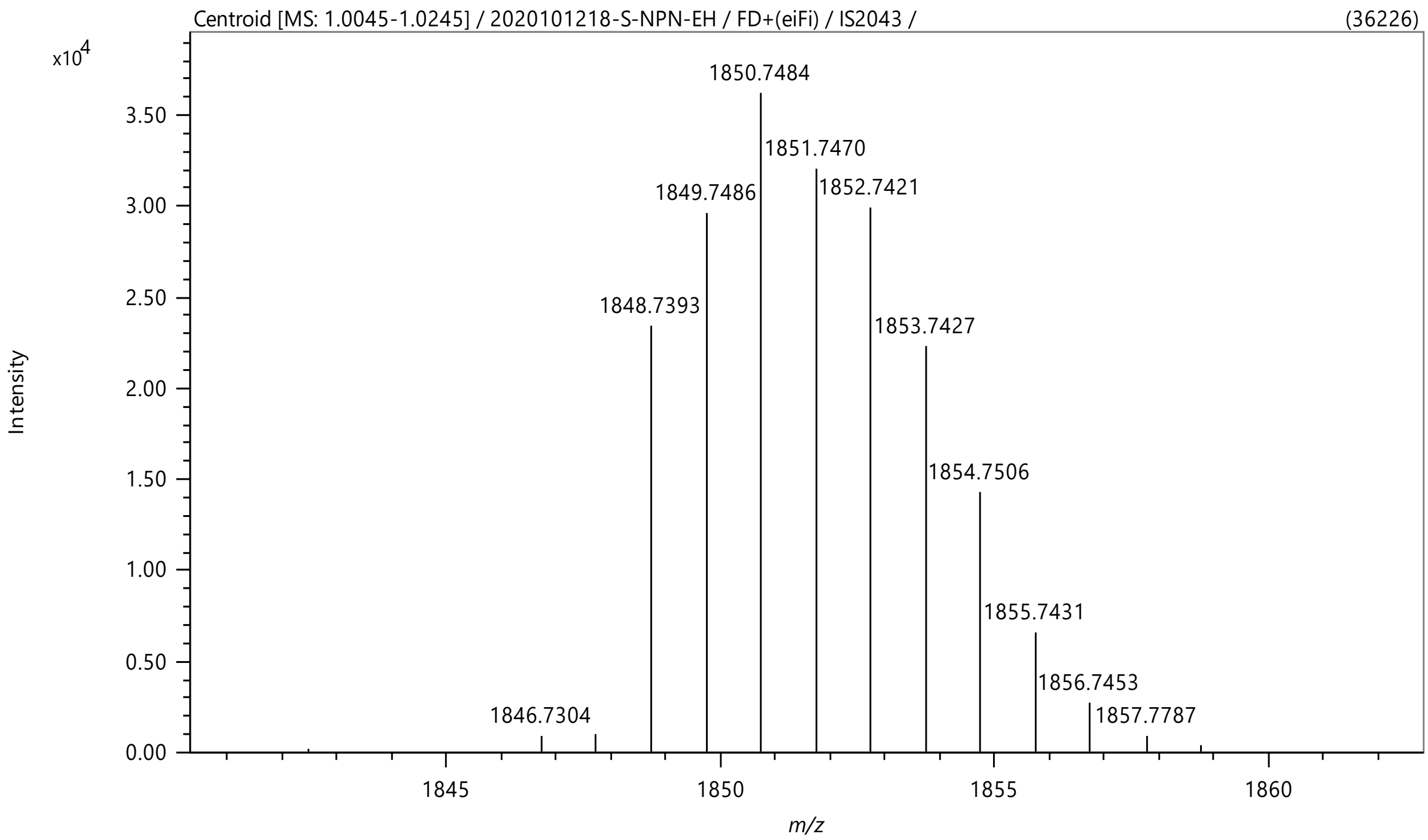

Figure S15. HR-FD mass spectrum of S-NPN-EH.

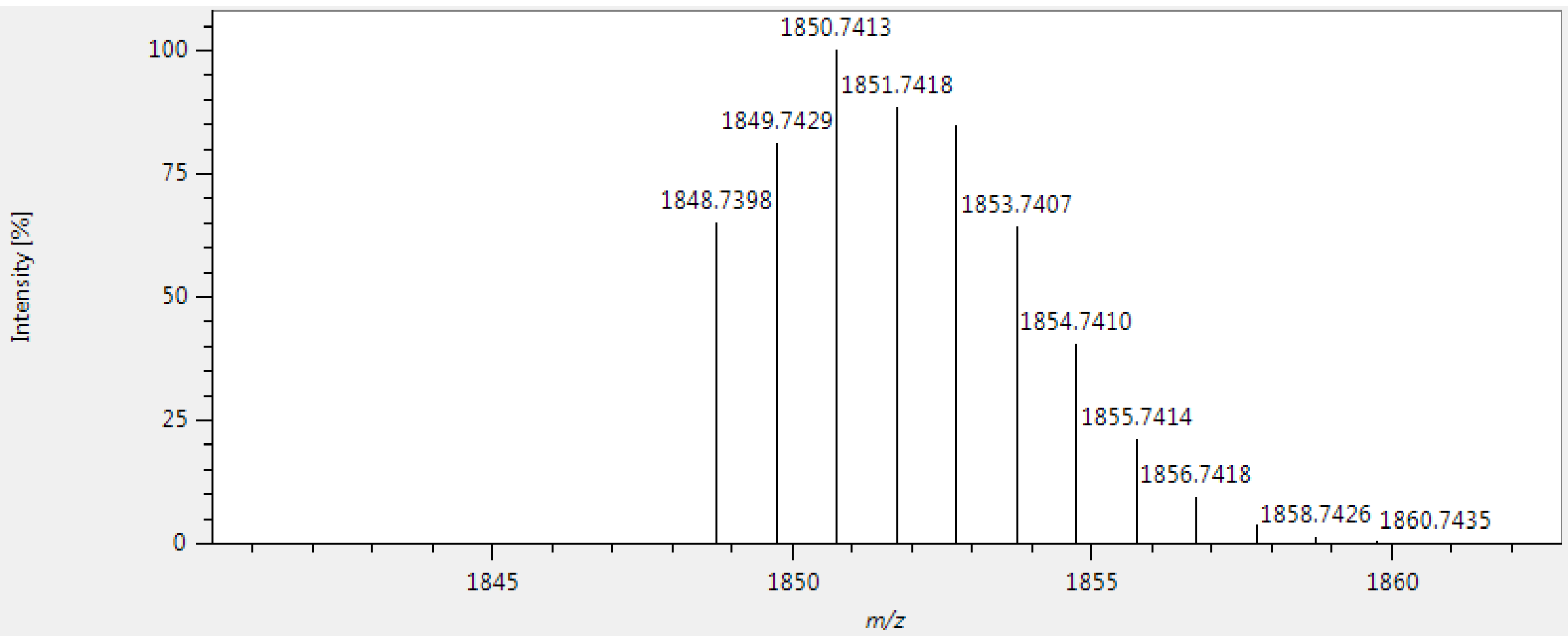

	Mass	Intensity	Formula	Calculated Mass	Mass Difference [mDa]	Mass Difference [ppm]	DBE
▶	1848.73934	23476.19	C108 H120 N8 O8 S4 Zn	1848.73979	-0.45	-0.25	53.0

Figure S16. HR-FD mass spectrum of S-NPN-EH.

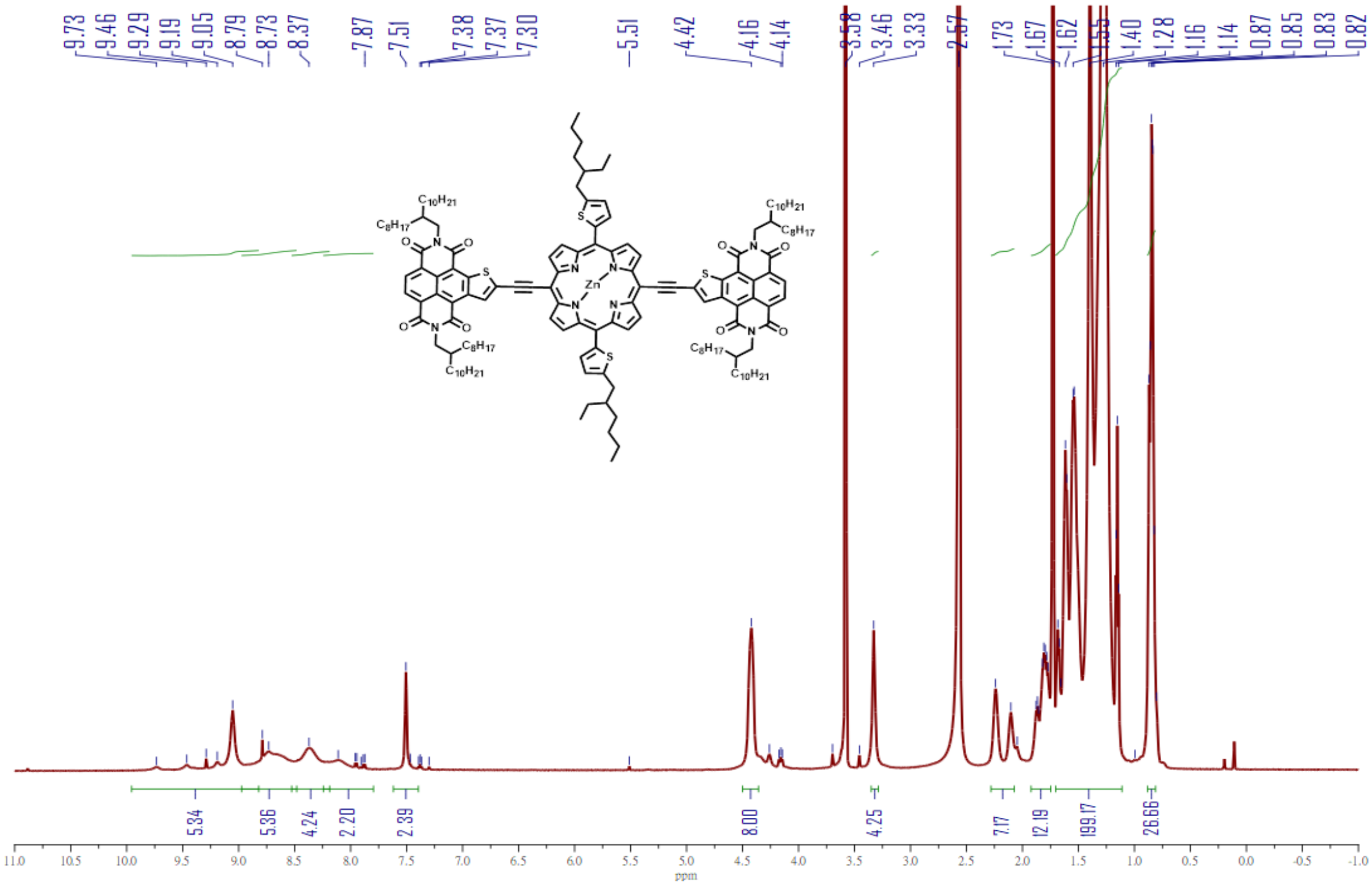

Figure S17. ^{1}H NMR spectrum of T-NPN-OD.

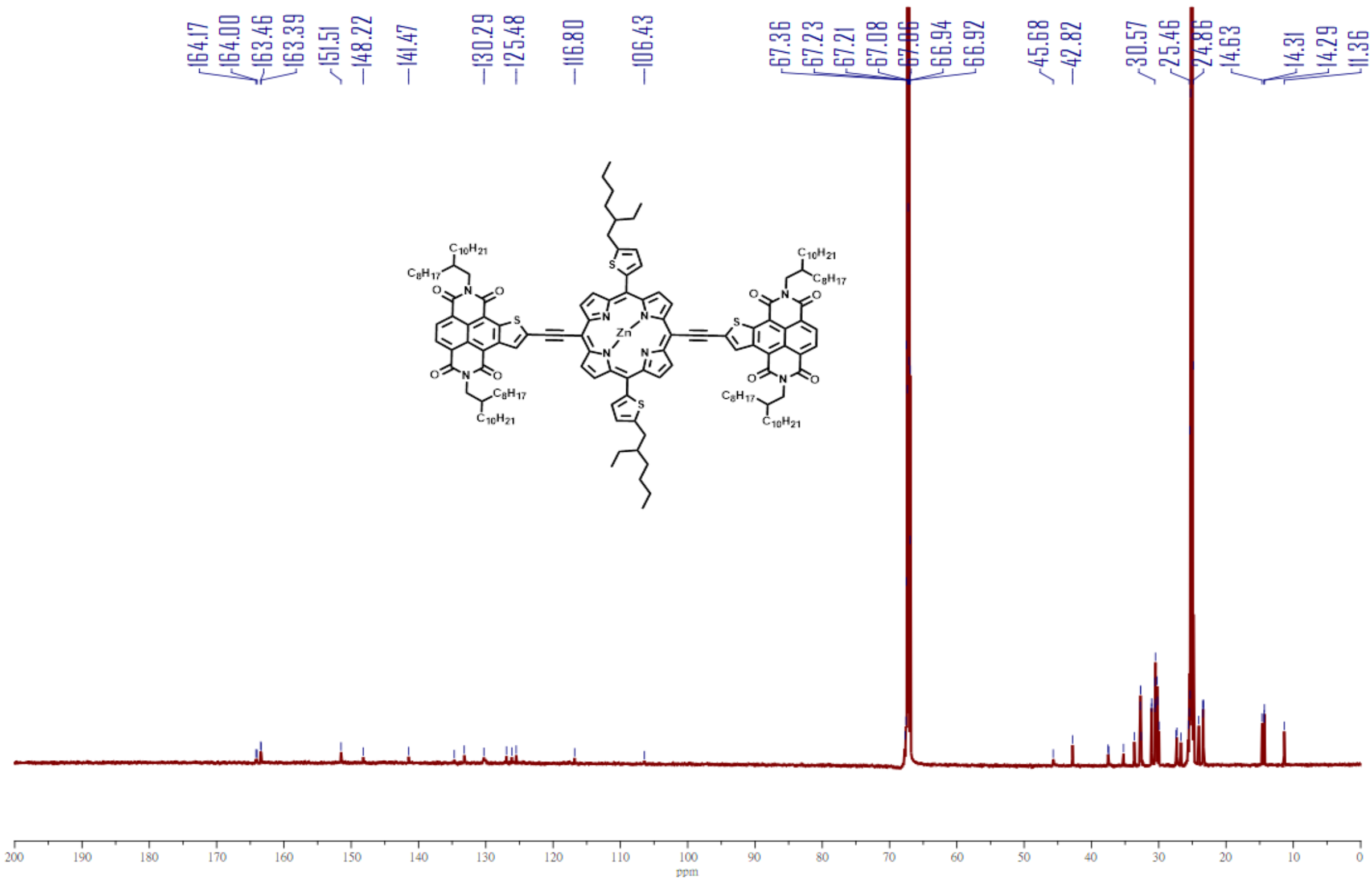

Figure S18. ^{13}C NMR spectrum of T-NPN-OD.

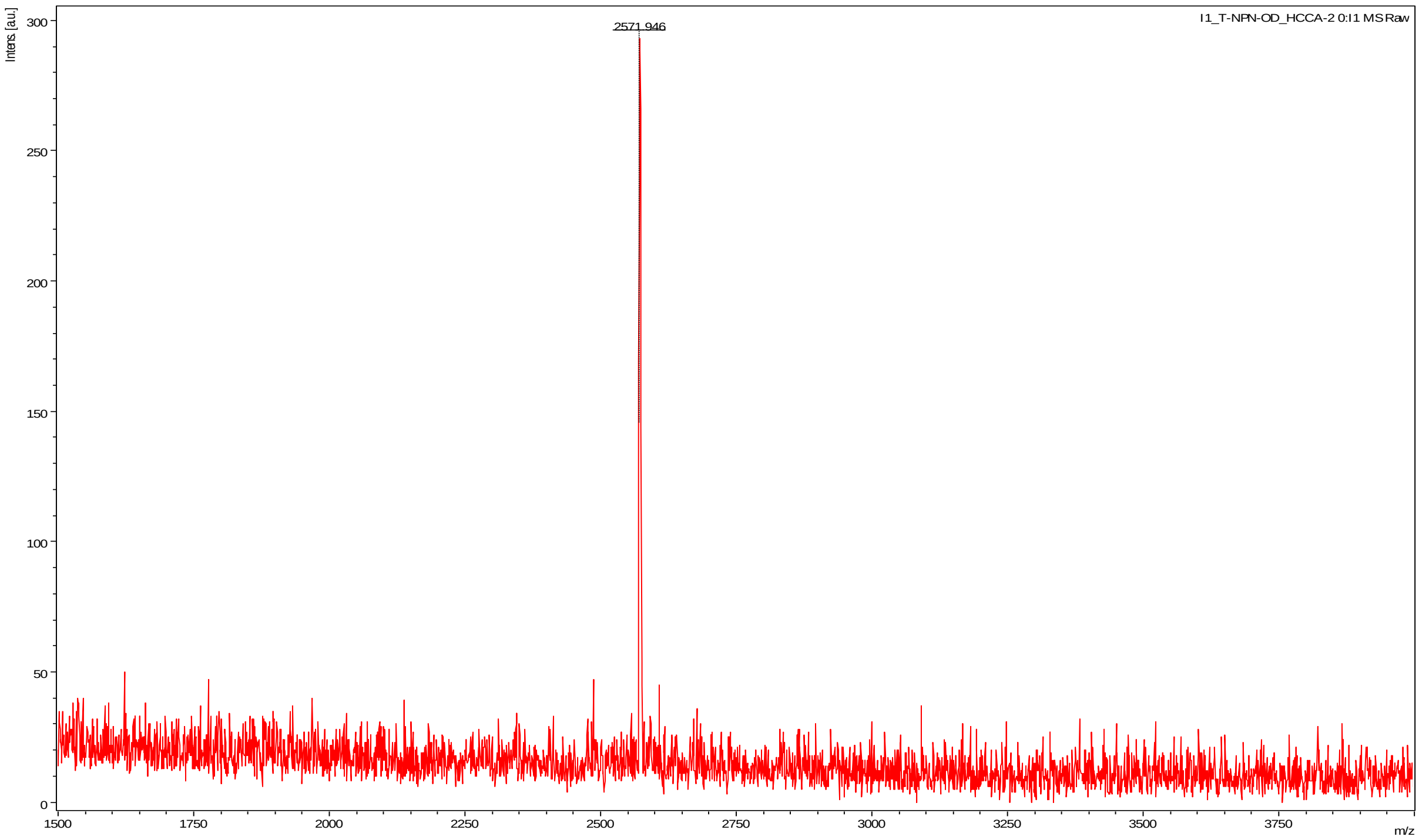

Figure S19. MALDI-TOF spectrum of T-NPN-OD.

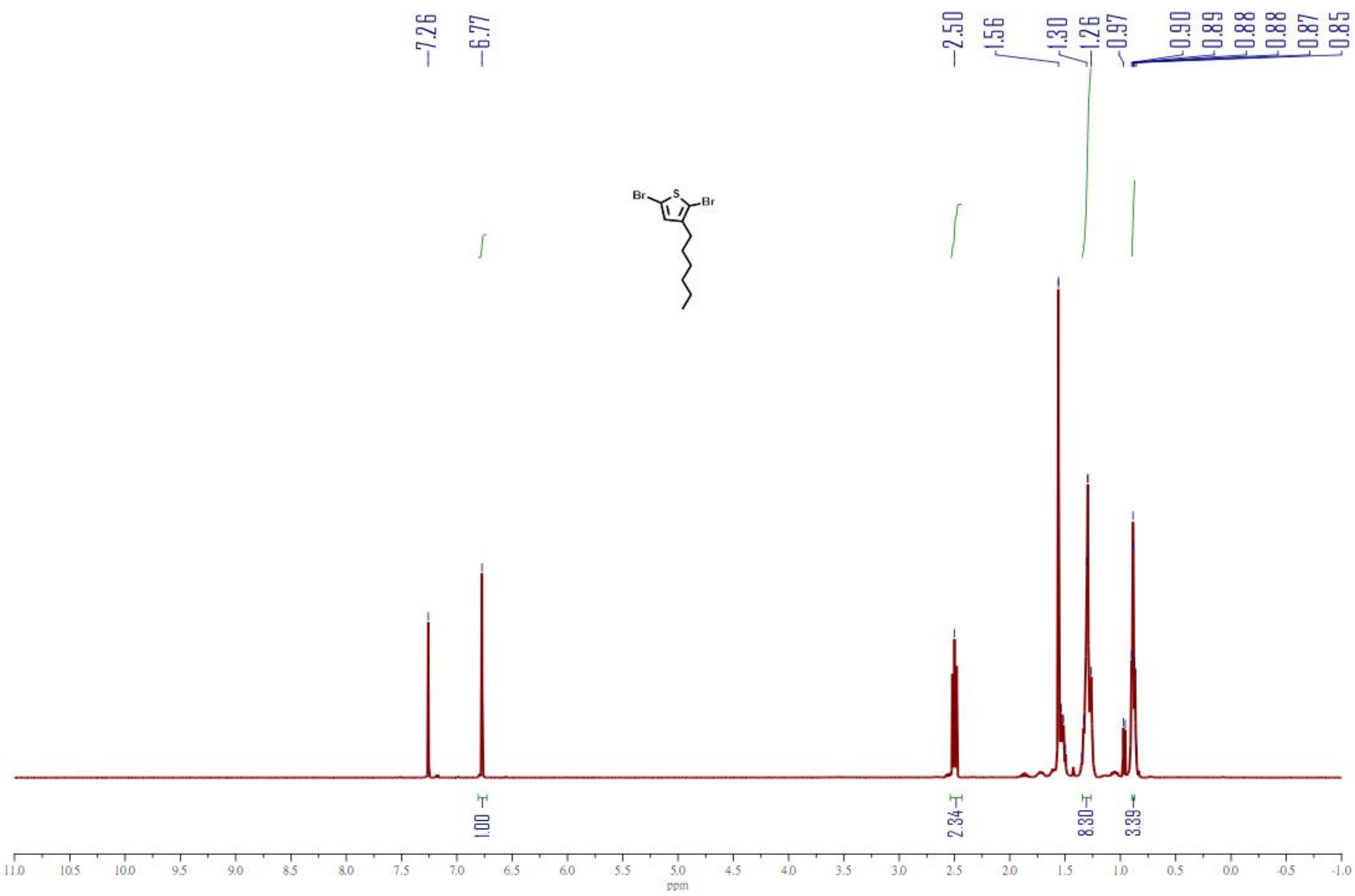

Figure S20. ^{1}H NMR spectrum of compound T1.

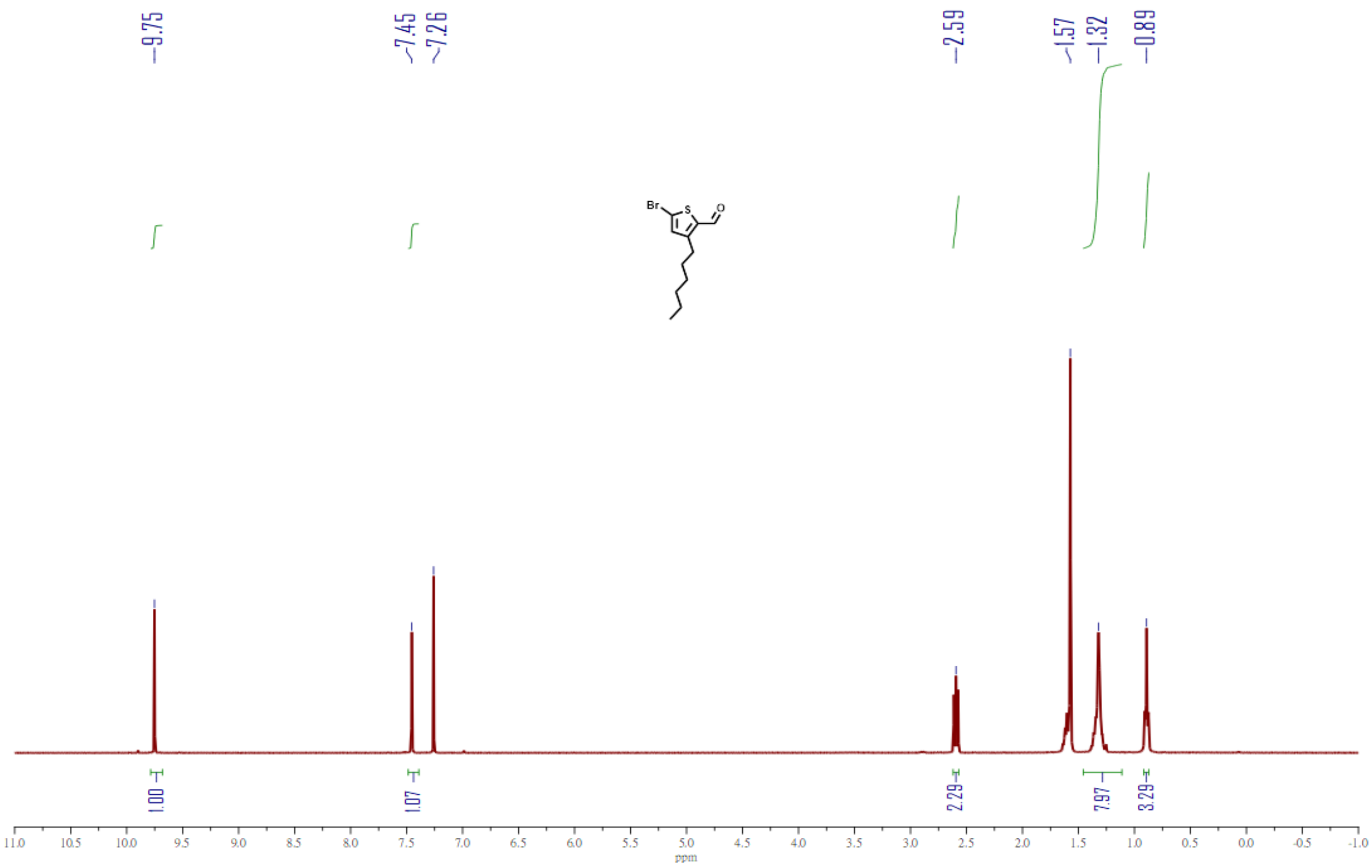

Figure S21. ^{1}H NMR spectrum of compound T2.

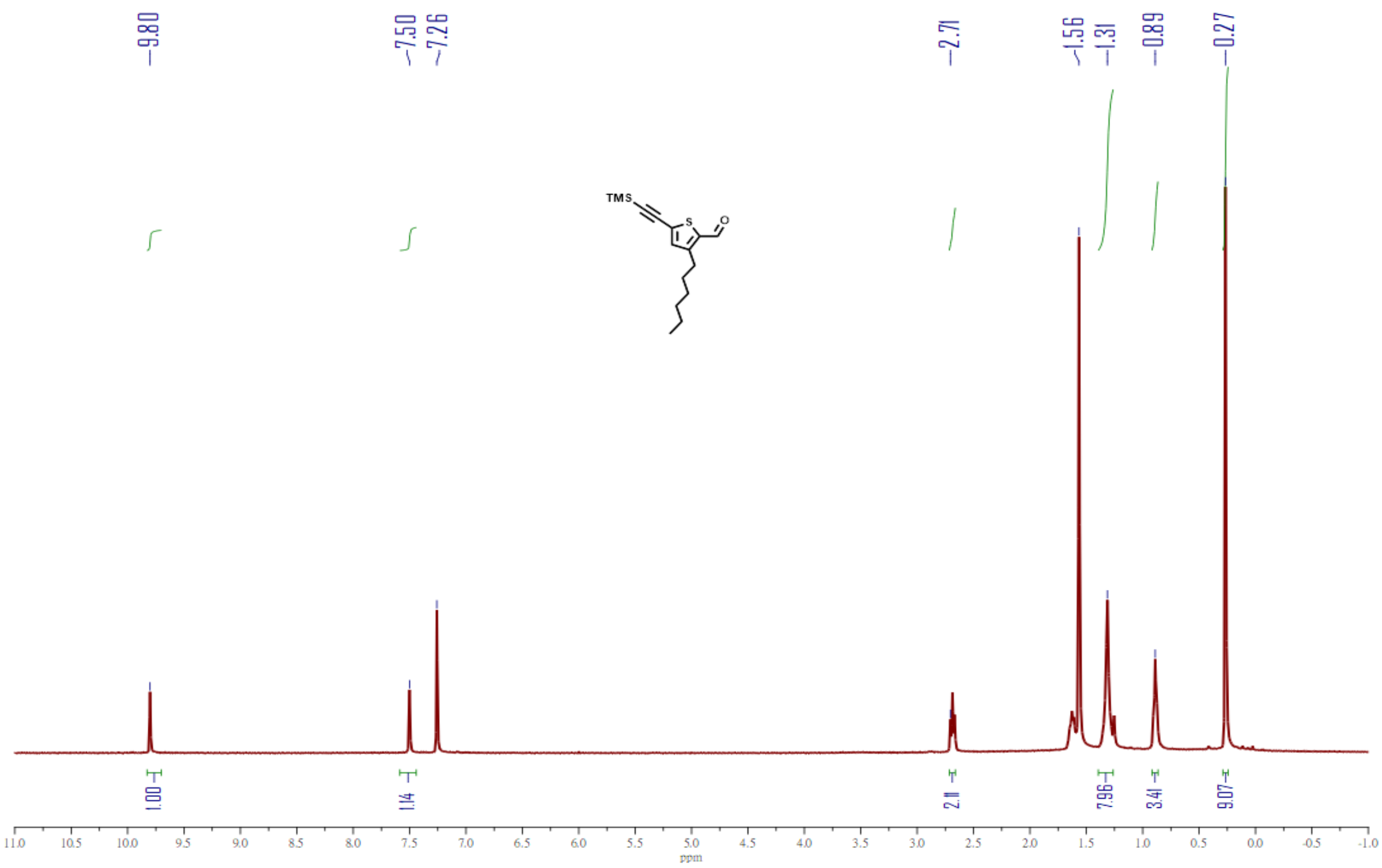

Figure S22. ^{1}H NMR spectrum of compound T3.

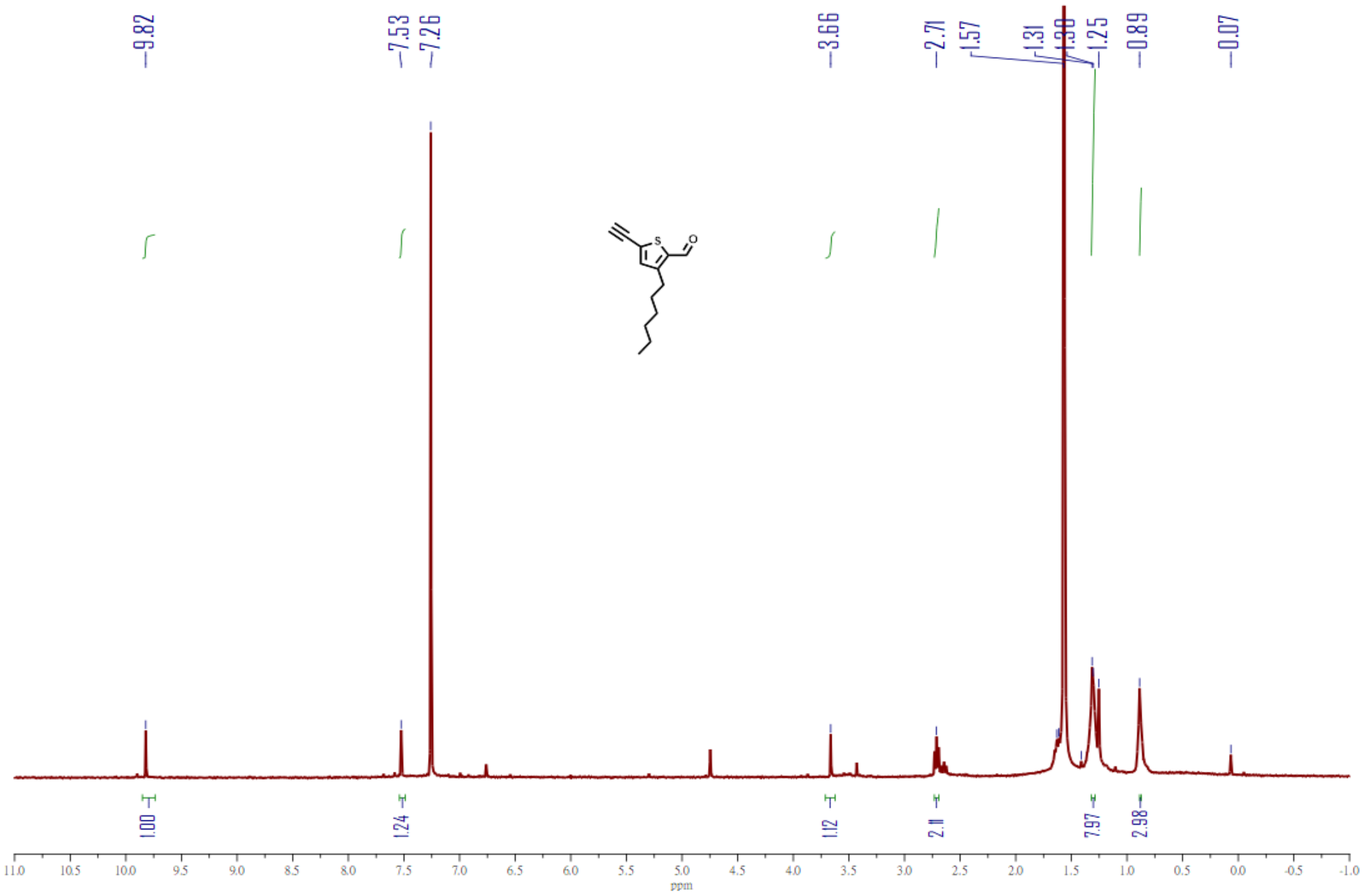

Figure S23. ^{1}H NMR spectrum of compound T4.

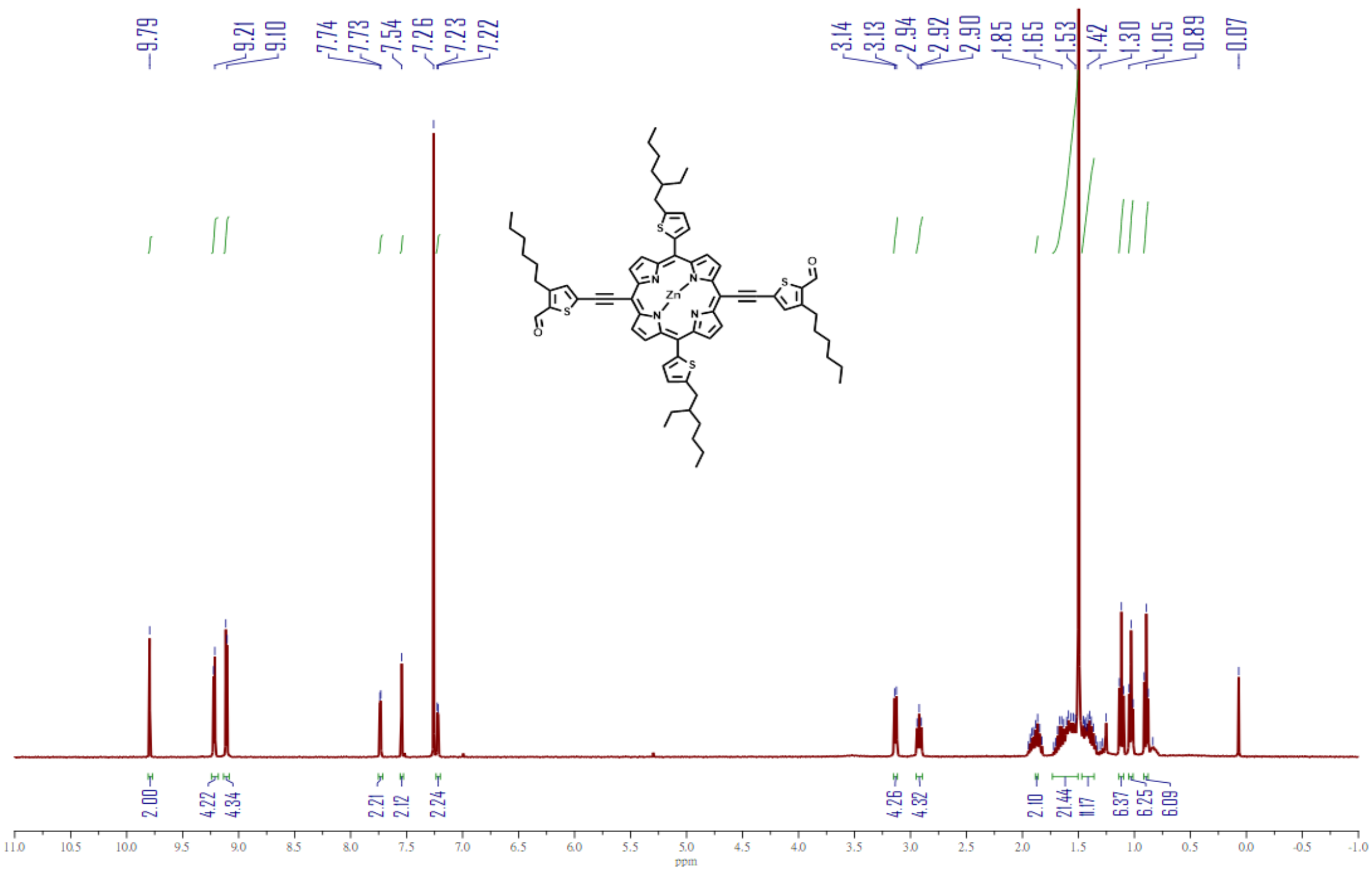

Figure S24. ^{1}H NMR spectrum of compound P5.

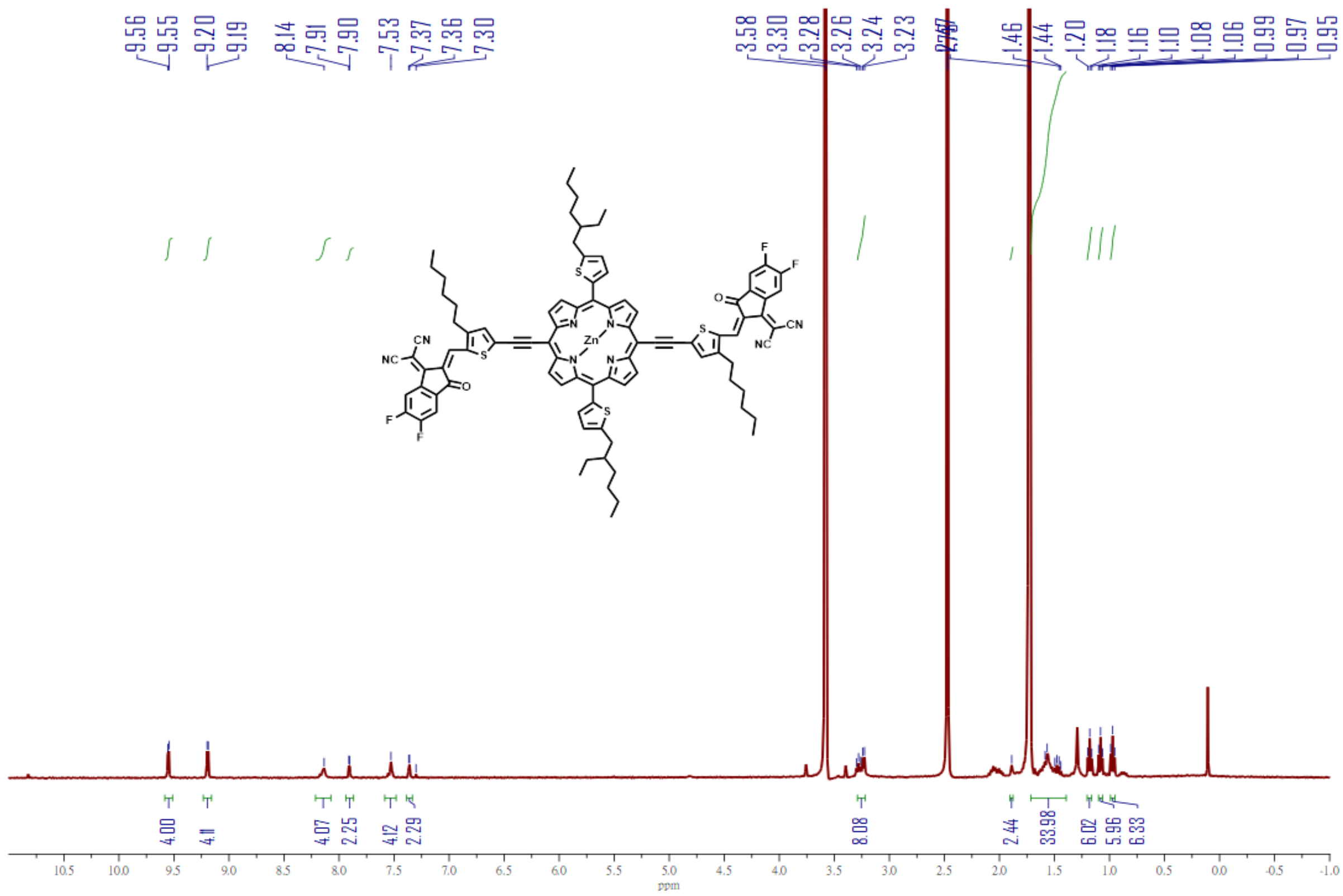

Figure S25. ^{1}H NMR spectrum of POR6-4FIC.

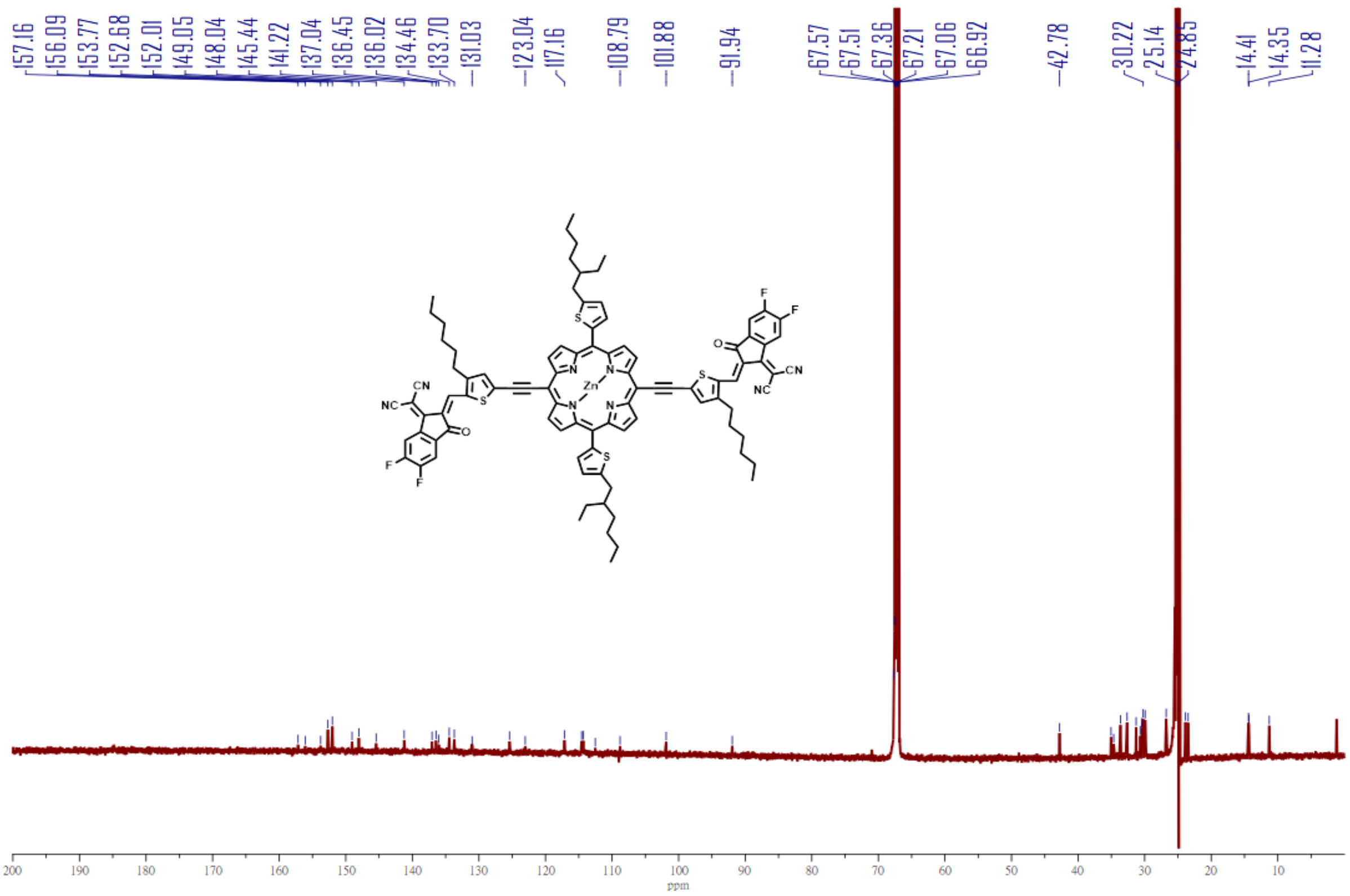

Figure S26. ^{13}C NMR spectrum of POR6-4FIC.

2. Thermal Properties

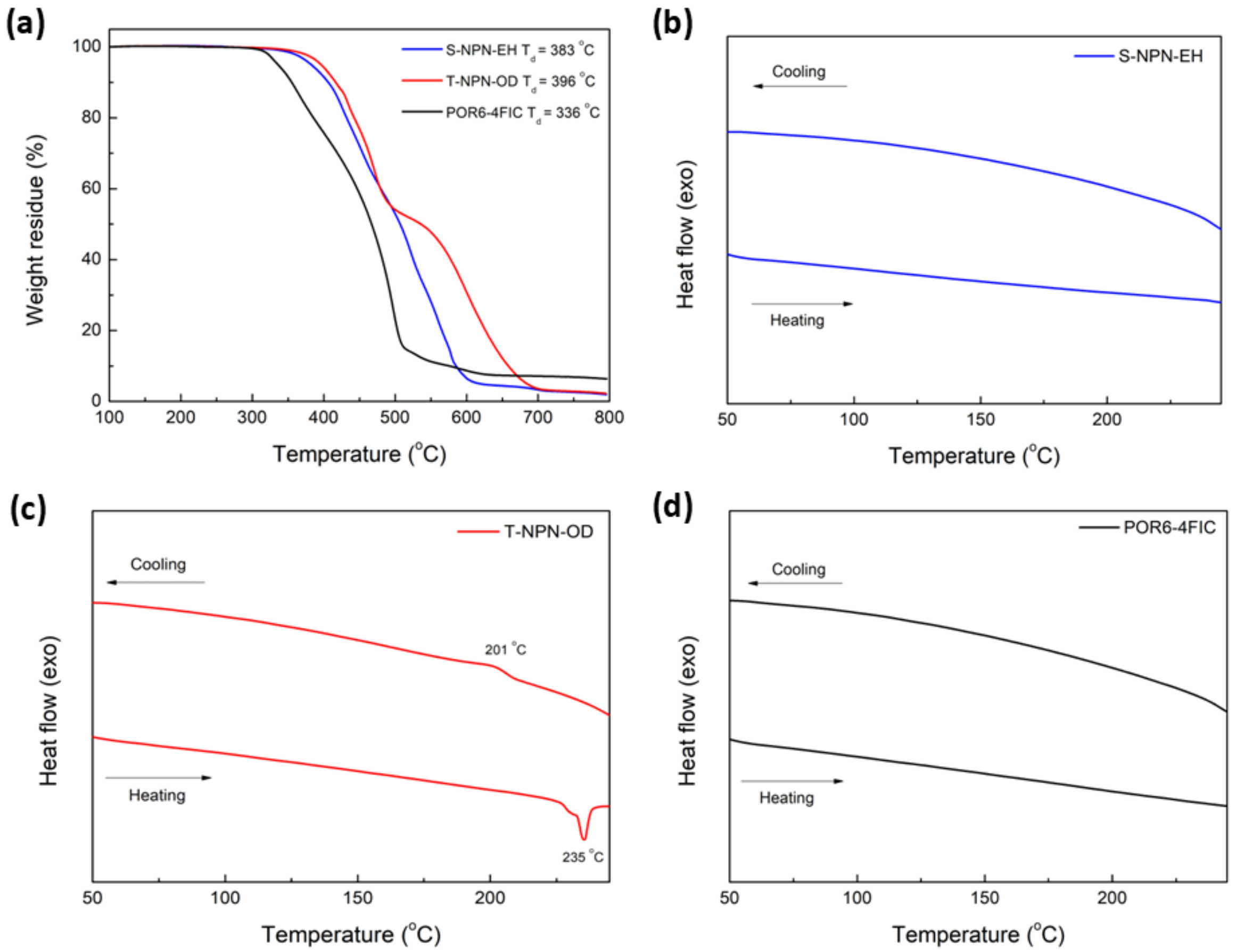

Figure S27. (a) TGA thermograms of S-NPN-EH, T-NPN-OD, and POR6-4FIC. The heating/cooling DSC thermograms of (b) S-NPN-EH, (c) T-NPN-OD, and (d) POR6-4FIC.

3. Cyclic Voltametric (CV) Properties

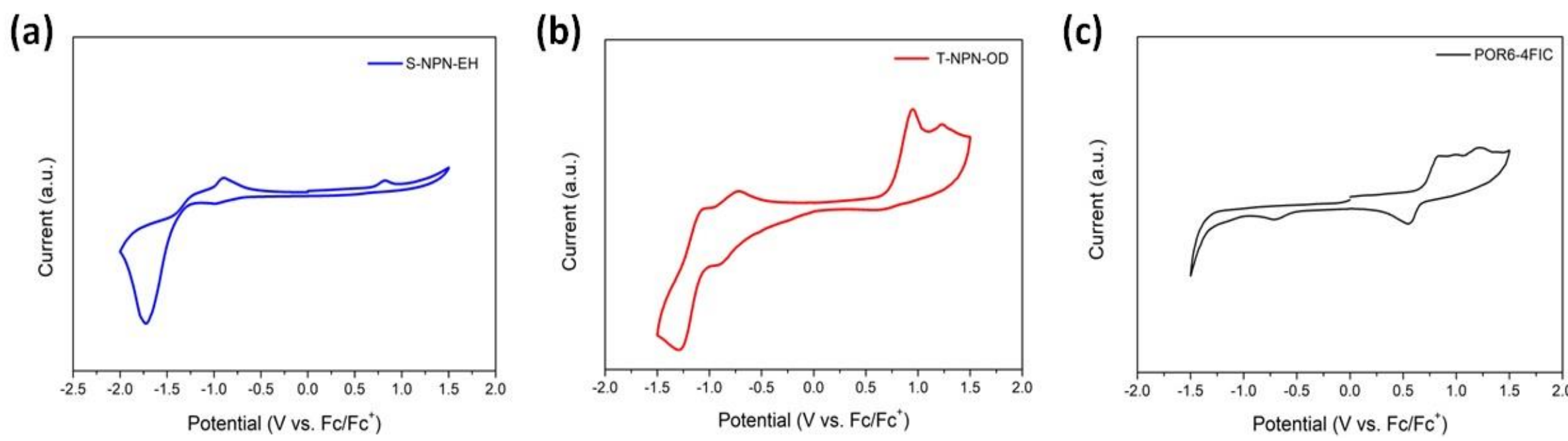

Figure S28. Oxidation/reduction plots of (a) S-NPN-EH, (b) T-NPN-OD, and (c) POR6-4FIC measured by CV.

4. DFT Calculations

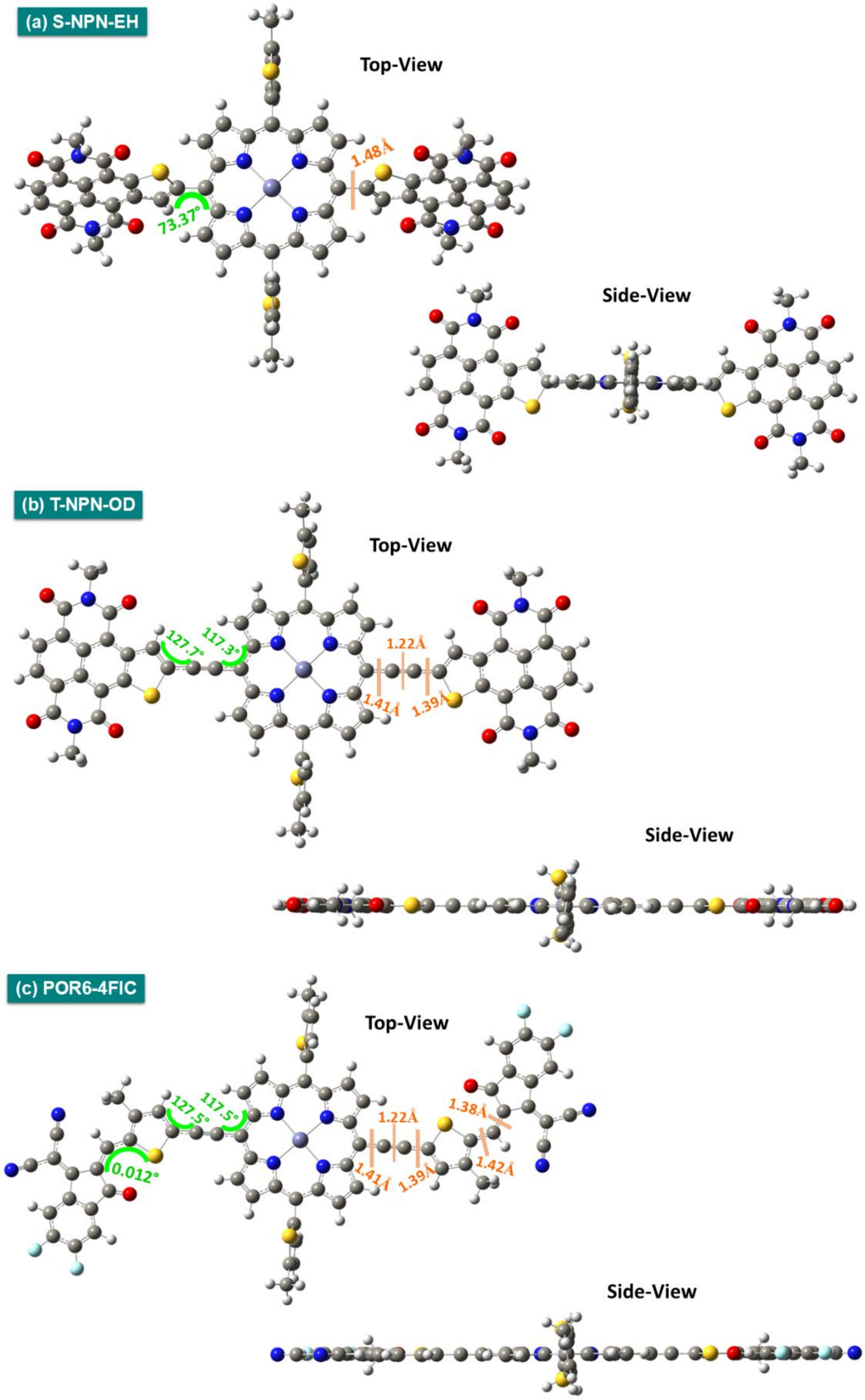

Figure S29. Optimized molecular geometries (top/side view) of (a) S-NPN-EH, (b) T-NPN-OD, and (c) POR6-4FIC.

5. BHJ-OSCs Device Characteristics

Table S1. Optimized photovoltaic performances of T-NPN-OD-based inverted BHJ-OSCs with various device fabrication conditions

Donor:Acceptor	Solvent/ Additive	Active-Layer TA	V_{oc} (V)	J_{sc} (mA cm^{-2})	FF (%)	PCE (%)
PBDB-T:T-NPN-OD (1:1.0)	CF/–	100 °C/10 min	0.75	2.23	41.81	0.71
PBDB-T:T-NPN-OD (1:1.0)	CF/–	–	0.76	2.73	40.32	0.82
PBDB-T:T-NPN-OD (1:1.0)	CF/Py-0.8%	130 °C/10 min	0.76	2.3	42.64	0.73
PBDB-T:T-NPN-OD (1:1.0)	CF/Py-0.8%	–	0.74	2.59	42.8	0.85
PBDB-T:T-NPN-OD (1:1.0)	CB/–	100 °C/10 min	0.74	3.22	42.53	1.01
PTB7-Th:T-NPN-OD (1:1.0)	CF/–	–	0.72	0.42	33.27	0.10
PTB7-Th:T-NPN-OD (1:1.0)	CF/–	135 °C/5 min	0.72	0.42	33.44	0.10
J71:T-NPN-OD (1:1.2)	CF/–	–	0.77	0.43	30.62	0.10
J71:T-NPN-OD (1:1.2)	CF/–	130 °C/10 min	0.73	0.49	29.0	0.10
J61:T-NPN-OD (1:1.2)	CF/–	–	0.79	0.3	32.27	0.08
J61:T-NPN-OD (1:1.2)	CF/–	130 °C/10 min	0.75	0.4	30.49	0.08
J61:T-NPN-OD (1:1.2)	CF/Py-1.0%	100 °C/10 min	0.8	0.03	27.61	0.01

Table S2. Optimized photovoltaic performances of POR6-4FIC-based inverted BHJ-OSCs with various device fabrication conditions

Donor:Acceptor	Solvent/ Additive	Active-Layer TA	V_{oc} (V)	J_{sc} (mA cm^{-2})	FF (%)	PCE (%)
PBDB-T:POR6-4FIC (1:1.2)	THF/–	130 °C/10 min	0.28	0.32	18.19	0.02
PBDB-T:POR6-4FIC (1:1.2)	THF/–	–	0.28	0.42	21.07	0.02
PTB7-Th:POR6-4FIC (1:1.2)	THF/–	130 °C/10 min	0.70	1.45	31.62	0.32
PTB7-Th:POR6-4FIC (1:1.2)	THF/–	–	0.71	1.28	32.0	0.28
PTB7-Th:POR6-4FIC (1:1.2)	CB/–	110 °C/10 min	0.74	0.86	28.2	0.18
PTB7-Th:POR6-4FIC (1:1.2)	CB/–		0.74	0.74	29.8	0.17

Table S3. Optimized photovoltaic performances of S-NPN-EH-based inverted BHJ-OSCs with various device fabrication conditions

Donor:Acceptor	Solvent/ Additive	Active-Layer TA	V_{oc} (V)	J_{sc} (mA cm^{-2})	FF (%)	PCE (%)
PBDB-T:S-NPN-EH (1:1.2)	DCB/Py-1%	130 °C/10 min	0.84	3.03	54.29	1.38
PBDB-T:S-NPN-EH (1:1.2)	DCB/Py-1%	–	0.84	2.91	51.3	1.25
PTB7-Th:S-NPN-EH (1:1.2)	CF/Py-1%	130 °C/10 min	0.79	5.98	41.46	1.95
PTB7-Th:S-NPN-EH (1:1.2)	DCB/–	130 °C/10 min	0.77	3.96	40.14	1.23
PTB7-Th:S-NPN-EH (1:1.2)	DCB/–		0.77	3.61	38.46	1.07
PTB7-Th:S-NPN-EH (1:1.2)	DCB/Py-1%	130 °C/10 min	0.8	5.69	48.15	2.19
PTB7-Th:S-NPN-EH (1:1.2)	DCB/Py-1%		0.84	3.0	51.47	1.30
